# Emergence of a Small-World Functional Network in Cultured Neurons

**DOI:** 10.1371/journal.pcbi.1002522

**Published:** 2012-05-17

**Authors:** Julia H. Downes, Mark W. Hammond, Dimitris Xydas, Matthew C. Spencer, Victor M. Becerra, Kevin Warwick, Ben J. Whalley, Slawomir J. Nasuto

**Affiliations:** 1 School of Systems Engineering, University of Reading, Whiteknights, Reading, Berkshire, United Kingdom; 2 School of Chemistry, Food Biosciences and Pharmacy, University of Reading, Whiteknights, Reading, Berkshire, United Kingdom; Indiana University, United States of America

## Abstract

The functional networks of cultured neurons exhibit complex network properties similar to those found *in vivo*. Starting from random seeding, cultures undergo significant reorganization during the initial period *in vitro*, yet despite providing an ideal platform for observing developmental changes in neuronal connectivity, little is known about how a complex functional network evolves from isolated neurons. In the present study, evolution of functional connectivity was estimated from correlations of spontaneous activity. Network properties were quantified using complex measures from graph theory and used to compare cultures at different stages of development during the first 5 weeks *in vitro*. Networks obtained from young cultures (14 days *in vitro*) exhibited a random topology, which evolved to a small-world topology during maturation. The topology change was accompanied by an increased presence of highly connected areas (hubs) and network efficiency increased with age. The small-world topology balances integration of network areas with segregation of specialized processing units. The emergence of such network structure in cultured neurons, despite a lack of external input, points to complex intrinsic biological mechanisms. Moreover, the functional network of cultures at mature ages is efficient and highly suited to complex processing tasks.

## Author Summary

Many social, technological and biological networks exhibit properties that are neither completely random, nor fully regular. They are known as complex networks and statistics exist to characterize their structure. Until recently, such networks have primarily been analyzed as fixed structures, which enable interaction between their components (nodes). The present work is one of the first empirical studies investigating the adaptation of complex networks [1]. Network evolution is particularly important for applying complex network analysis to biological systems, where the evolution of the network reflects the biological processes that drive it. Here, we characterize the functional networks obtained from neurons grown *in vitro*. Network properties are described at seven day intervals during the neurons' maturation period. Initially, neurons formed random networks, which spontaneously reorganized to a ‘small-world’ architecture. The ‘small-world’ concept derives from the study of social networks, where it is referred to as ‘six-degrees of separation’: the connection of any two individuals by as few as six acquaintances. In brain networks, this translates to rapid interaction between neurons, mediated by a few links between locally connected clusters (cliques) of neurons. This architecture is considered optimal for efficient information processing and its spontaneous emergence in cultured neurons is remarkable.

## Introduction

The organizational properties of biological, technological and social systems are increasingly being characterized by representing them as abstract networks of interacting components and quantifying non-random features of their structure [2], [3], [4], [5], [6]. Many real-world networks have an organization (topology) that is neither completely random, nor completely regular. Termed complex networks, these typically afford excellent integration between their constituent parts yet they also provide tightly interconnected subnetworks that segregate efficient within-group interaction. An example is social networks, for which a seminal study [7] revealed that any two individuals in the world could communicate via only a small number (∼6) of mutual acquaintances. Such networks are sparse – only a tiny proportion of the world's population are associated, yet they are incredibly well-connected. The phenomenon has been termed ‘small-world’ – hence the concept of a small-world network.

For neuronal connectivity, the abstract network (graph-theoretic) approach to analysis has allowed common organizational principles to be identified at both the macroscale level of whole brain imaging [8], [9], [10], [11], and the microscale level of connections between individual neurons [12], [13]. Importantly, this form of analysis enables the relationship between neuronal network organization and (whole or partial) brain function to be investigated. There are numerous complex network statistics for assessing the non-random properties of these abstract networks (for review see [14]), each of these statistics enable direct comparison of results from diverse experiment modalities and over a range of species and scales [2], [5]. Moreover, properties may also be compared with those of networks from other domains [15]. Two important metrics are the level of integration and segregation; high levels of which are found in random and lattice networks, respectively. Since small-world networks have high levels of both properties, the extent to which a given network approximates or deviates from small-worldness may be evaluated by considering the balance between integration and segregation [16], [17]. This balance has become an important benchmark for the assessment of neuronal networks and the small-world topology has been found at multiple scales over a range of species in both structural [16], [18] and functional [9], networks. Moreover, its influence on network efficiency [8] and robustness [9] has been demonstrated, and deviation from the small-world topology has been associated with abnormal or decreased brain function [8], [19], [20], [22], [23].

The focus of the present study is the development of complex network properties within cultures of neurons, grown *in vitro*. Unlike *in-vivo* brain networks, where the range of experimental conditions is typically constrained by the availability of subjects with a given condition, or strict regulation regarding experimental manipulation, cultures of dissociated neurons grown on multi-electrode arrays (MEAs) provide an experimental platform for the long-term investigation and manipulation of neuronal cells. Such neurons spontaneously form connections [24], [25] and non-random properties have been found in the resulting structural network [13]. Moreover, cultures share several important characteristics with their *in vivo* counterparts [26], [27], [28], for review see [25]. Consequently, these preparations are increasingly being used in investigations of cellular and network processes that underlie complex cognitive functions [29], [30], [31], [32], [33] and as models of pathophysiological states (e.g. epilepsy and stroke [34]). Importantly, since the cultures have no pre-built infrastructure, they allow the network formation to be observed - making them well-suited to investigating neuronal network development in a living biological system.

### Cultured neurons and investigating network function

Two aspects of the cultures that are of particular interest are their structural (anatomical) circuitry and the interactions which take place over this circuitry, both determining the computational capacity of the underlying network. Whilst cultures are typically too dense for accurate observation of their structural connectivity, analysis of functional connectivity provides a probabilistic estimation of the relationship between distributed neuronal units [2], thereby enabling spatio-temporal interactions between areas of the network to be measured throughout experiments. This provides a useful means to investigate the network properties of cultures, particularly since functional connectivity estimated over certain timescales may contain information about the underlying structural network [35].

Existing literature indicates that the functional network properties of cortical cultures change during maturation [36] and following stimulation [29], [37], [38]. However, such studies have focused on changes in the expected link-level properties such as the mean strength and metric distance of connections [36], or the proportion of links which are strengthened or weakened following stimulation [37], [38]. These aggregate measures capture gross changes in global connectivity, but they do not reflect the organizational features of the network, e.g. the distribution of properties amongst the neural units, or whether there are groups of neural units that are more densely connected than others. Analysis of such organizational features would reveal the architecture of the network, enabling investigation into which interactions the network could support and how the network organization changes under different experimental conditions. Importantly, by assessing the complex network properties, the relevance of results from cultures to investigations of whole-brain networks would be increased.

Reports that rigorously compare culture's complex network properties under different experimental conditions are very sparse. Mature cultures were assessed in [39] and networks from cultures subject to an *in vitro* glutamate injury model of epileptiform activity were assessed in [34]. The utility of cultures for investigating changes in cognitive function, characterizing drug effects and modeling disease states, could be greatly extended by applying complex network statistics to quantify the influence of experimental manipulation on the network architecture. Moreover, comparison with results from *in vivo* networks may reveal basic organizational principles common to both.

Experiments utilizing cultures can be undertaken across a range of ages, yet little is known about whether developmental changes occur in culture's complex network properties. Questions such as when and which non-random properties are present, their stability over time and the variability between cultures and their ages remain largely unanswered. The nature of such spontaneously occurring changes in a culture's functional network are important *a priori* knowledge for assessing experimental outcomes using complex network statistics. Moreover, by analyzing these ‘known’ conditions, a framework can be established for evaluating a variety of experimental conditions, including those resulting from embodying a culture in a closed loop system. [40], [41], [42], [43].

The density at which cultures are seeded exerts an important influence on the rate of maturation. Dense cultures mature faster than their sparse equivalents, and they demonstrate bursting activity earlier in development [44]. For the purpose of the present paper, dense cultures were deemed preferable, since their use enabled network properties to be measured earlier in development than would have been possible on much sparser cultures. Additionally, to investigate changes in the *functional* network properties during culture maturation, maintaining consistency in plating parameters was important to minimize differences in cultures structural properties. Such differences would have complicated the analysis and interpretation of results. Therefore, cultures at a fixed density were used (those described in [44] as ‘dense’). At ∼1,500 to 6,500 cells within the ∼1.6 mm^2^ recording area of the MEA, the cells in such cultures form a monolayer. Moreover, they can be maintained for many months [45] and the density is comparable to that used by other groups (typically ∼2,500–3,000 cells per mm^2^ [24], [30], [36], [42], [46], [47]).

The present study establishes the baseline network statistics for cultures at specified stages of development and uses them to characterize culture maturation. The topological, spatial and performance properties of functional networks captured every 7 days (7 to 35 days *in vitro* [DIV]) were compared using a population of 10 cultures. The study is one of the first to investigate functional connectivity in an evolving complex system. Here, the evolution of network properties is a counterpart of biological processes shaping the culture's development.

### Methodological considerations

Since the graph-theoretic approach and use of complex network statistics is a relatively novel method for investigating functional connectivity in cultures, the key methodological decisions are described next.

#### Applying network connectivity analysis to multi-electrode array data

**Figure 1:**
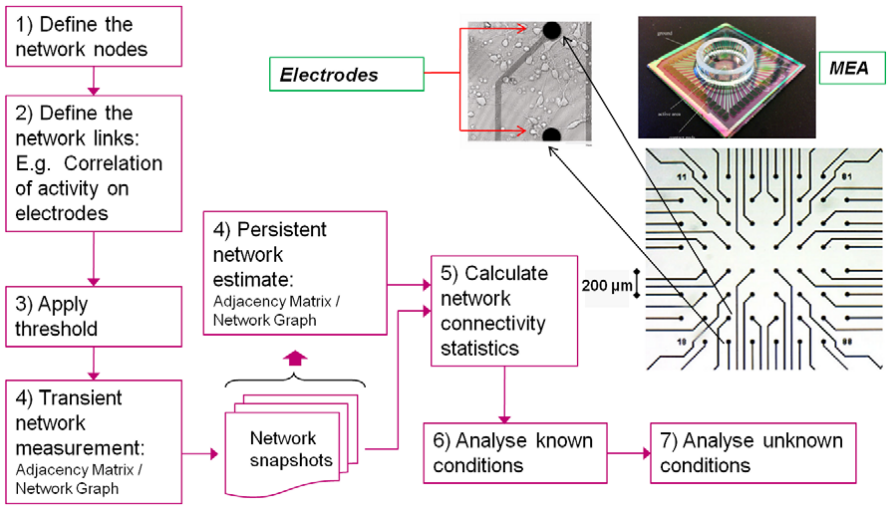
Steps in functional connectivity analysis of multi-electrode array data. Steps 1–3 and step 5 are based on recommendations from Bullmore & Sporns (Nature Reviews Neuroscience, 2009). Steps 4, 6 and 7 refer to techniques specific to analysis of culture activity recorded from multi-electrode arrays (MEAs, example pictured top right). The 8×8 grid indicates the recording area of the MEA (inset: close-up of two electrodes with visible neurons in their vicinity).

Although both structural and functional neuronal networks can be explored using graph theory [5], [14], the present study concentrates on functional networks. The main steps involved in a graph-theoretic analysis of neuronal networks are described in [5]. Figure 1 illustrates their application to neuron cultures (or other *in-vitro* preparations utilizing multi-electrode recordings). At step 1, the nodes of the network are defined: for the present study, potential nodes were the 59 electrodes (channels) of the MEA. At step 2, functional links are defined, for example using activity recorded from the electrodes: a computationally straightforward technique estimating pair-wise correlation of spike-times recorded via MEA electrodes [34] was used for the links herein. Many techniques exist to estimate dependence between time-series [48], [49] and, whilst the choice is in the hands of the experimenter, the decision may influence the interpretation of results. Regardless of the chosen technique, it is important to consider the time-period over which links are estimated, particularly with respect to the form of activity that will be used to define inter-node connections. Due the non-stationary mixture of high frequency bursting and low-frequency ‘tonic’ activity found in cultures [44], the links herein were defined over two time-scales:

Firstly, at short time-scales (hundreds of milliseconds), connectivity was assessed during each network-wide burst, a threshold was then applied to include only the links between highly related nodes [5] (Step 3). Secondly, to filter out inter-burst fluctuations in activity levels, the ‘persistent’ network infrastructure was estimated over a longer time- scale (20 minutes) based on the frequency with which links were identified over the set of burst-based (‘transient’) networks (step 4). The application of a threshold at step 3 reduces the complexity of the analysis, and is useful if link ‘strength’ is not the focus of the study. However, selection of an appropriate threshold is important for the interpretation of results.

To compare networks from a sequence of experimental ‘conditions’, the development of the *network* itself may be of equal importance to the development of its topology. However, methods for characterizing functional connectivity principally focus upon static networks. Analysis of networks evolving over time is more challenging; network evolution involves the birth and death of links and in some cases, nodes themselves. Consequently, it is not desirable to fix the number of nodes, or adjust the link definition threshold to achieve a pre-determined connection density (c.f. [9], [20]). Thus, for the present study, a relative threshold (based on the specificity of the cross-covariance peak) determined whether a given link was included in the network or regarded as ‘noise’. Once all potential links had been assessed, only those nodes with a connection to at least one other node were included in the network.

The dual time-scale approach to network definition (Figure 1, Step 4) results in two types of network graph, which enables assessment of a culture's network activity over different timescales. For the work herein, analysis of the topological and non-topological properties of the longer time-scale persistent networks allowed structural and spatial properties to be investigated every 7 DIV, thereby characterizing the network development. Additionally, at short time-scales, the set of each cultures' transient networks allowed the activity that took place over the networks to be analyzed. This enabled performance and reliability metrics to be estimated.

A number of topological metrics may be calculated and from these the complex network statistics [2], [5], [14], [16], [18] may be derived (see Materials and Methods). In order to compare the persistent network properties at each age, both local (node related) and network-wide statistics were calculated. Table S1 provides definitions of all complex network measures used, many of which were described in [14]. The magnitude of the topological properties from a given network are dependent on the number of nodes (*n*, referred to herein as network ‘size’), the number of links (*m*) and the resultant edge density (*ξ*). To calculate complex network statistics, empirical network properties are compared against those expected in equivalent random (or lattice) null hypothesis networks [14]. These comparison networks have the same number of nodes and links, thus the same connectivity density. However, it is important to verify certain assumptions regarding the size and density of networks that may be compared (see Materials and Methods). Whilst the number of nodes (*n*) and the average number of connections per node (*K*) are often used to specify a graph's basic properties, this does not allow instant evaluation of edge density. Therefore, for the analysis herein, edge density was used instead of mean node degree. The property equates to the mean node degree normalized to the maximum possible, which provides a density measure (*ξ*) that is independent of the network size (*n*). The measure reflects the sparseness or ‘cost’ of the network [8] and most importantly, it can be directly compared between networks with different numbers of nodes.

Asides from the level of integration and segregation, an important aspect to characterizing a network's ‘class’ is the form of the degree distribution. This may be measured by determining the best fitting model: A fast-decaying (exponential or Gaussian) model provides a good fit for networks with a homogeneous population of nodes, whereby most nodes have a comparable number of connections and few nodes deviate from this number significantly. Such networks are classed as ‘single-scale’ and their scale is equal to their mean node degree. Conversely, networks that have no characteristic scale are termed ‘scale-free’, these are identified by a degree distribution that decays progressively more slowly towards infinity – hence there is no characteristic mean node degree. These are typically represented by a power-law model.

Random and lattice networks both have a single-scale degree distribution, conversely, many real-world networks have been found to possess a power-law degree distribution [50]. Since both single-scale [51] and power-law [52] degree distributions have been reported for neuronal networks, to ascertain the degree distribution of the networks herein, both exponential and power-law models were fitted to the data (see Materials and Methods). The ratio of goodness-to-fit values at each age was used to determine whether the distribution changed during the stages of maturation.

Since cultured neuronal networks are embedded in physical space, spatial and temporal characteristics of interaction, such as inter-node distance and signal propagation speed, can also be informative about changes in the activity patterns. Therefore, physical link length (derived from inter-electrode distance), and network-wide signal propagation efficiency (via mean burst propagation time) were also assessed. Additionally, the frequency with which individual links are activated can provide information on the influence of a given link in the various network interactions. Therefore, the reliability of link activation was calculated from the analog (weighted) persistence adjacency matrix. Tables 1 & 2 provide an overview of the main measures used for the present study, along with their range and interpretation.

**Table 1:** Topological & non-topological network properties for the present study (part 1).

Property type, name and Description			Range & units	Interpretation
**Complex network properties**	*L*	Mean path length: *L_Norm_*	1+ (# hops btw nodes)	Integration: Ability for any two nodes to interact via a minimal number of intermediary nodes. A short (low) mean path length reflects high integration (i.e. a low average number of hops between nodes) as found in random networks.
	*CC*	Mean clustering coefficient: *CC_Norm_*	0–1	Segregation: Ability for groups of nodes to interact. A high level of segregation (as found in lattice networks) reflects the presence of highly interconnected node subgroups (clusters) within the network.
	*S/W*	‘Small-worldness’: *CC_Norm_*/*L_Norm_*	0+	Complexity: balance between integration and segregation
**Non-topological properties**	Network broadcast time	Measured as the burst propagation time	∼100–1000 ms	Performance of the network in terms of the time required for a signal to reach all nodes

The measures used to quantify the persistent, and transient (last 1) network properties.

**Table 2:** Topological and non-topological network properties for the present study (part 2).

Property type, name and Description			Range, units and Interpretation
**Complex network properties**	Node degrees	Node degree distribution	Relative influence of nodes in the network (node degree = 1–58) -Nodes with a high degree have many connections: A fat tailed degree distribution indicates presence of highly influential nodes, whilst homogeneity indicates lack of network structure.
**Non-topological properties**	Link lengths	Spatial properties: Link-length distribution	Assess form and the proportion of links between nearby *vs* distant nodes (link length = ∼200–1980 µm). Metabolic cost increases with link length, short links incur lower cost.
	Link persistence levels	‘Reliability’ props: Link activation frequency distribution.	Assess form and the contribution of persistent links (link persistence = 0–1). Persistent links represent frequent interactions between neural units.

The measures used to assess the persistent, and transient (last 2) network properties (part 2).

## Results

Results are split into two sections. The first presents topological, then spatial network statistics from persistent networks. The second presents statistics on the propagation of activity over the network (from the transient networks). Network statistics were obtained for each culture at each age (DIV 7, 14, 21, 28, 35). NOTE, at DIV 7 only one culture was found to have a persistent network, therefore this age was not considered for the significance testing.

### Culture's persistent networks acquire non-random properties during development

**Figure 2:**
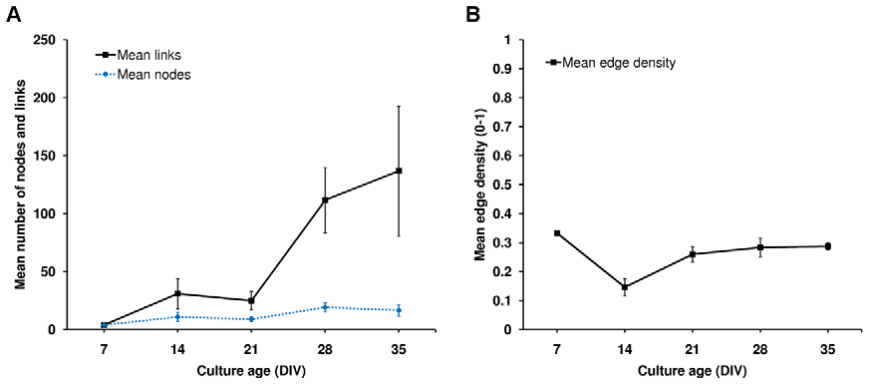
Basic topological properties of the persistent networks as a function of culture age. Number of nodes, links and edge density; calculated for 10 cultures at each age (DIV). Left: mean number of nodes and links found in the persistent networks. Note, although the number of nodes is a very different magnitude from the number of links, number of nodes was not found to change significantly (P = 0.272). Results for numbers of links at each age suggested an increase between younger (DIV 14 and DIV 21) and older (DIV 28 and 35) ages, however the increase was not significant (P = 0.074). Right: mean edge density of the persistent networks. Edge density (i.e. link density) quantifies the ‘cost’ of the network in terms of the number of links (*m*)/the maximum possible number of links ((n*(*n*−1)), given the number of nodes (*n*). Edge density was first calculated for each culture and then averaged over all cultures. Mean edge density at DIVs 21 to 35 was significantly higher than at DIV 14 (P = 0.012). In cases where no links were found the data were excluded from the analysis. All statistics quoted are for the *n* = 5–8 cultures valid for complex network analysis. Error bars represent ± standard error of mean (s.e.m, *n* = 5 to 8).

The number of nodes and links for a given culture was used to calculate the edge density of its network. Figure 2 shows the expected values for each property. The mean number of nodes was relatively constant and independent of age (P = 0.272). In contrast, the mean number of links measured at DIVs 14 and 21 was lower than at DIVs 28 and 35, with a strong trend towards a significant increase between the younger and older ages (P = 0.074). Edge density increased significantly between DIVs 14 and 21 (P = 0.012) and showed no significant change thereafter. Statistics quoted are for the *n* = 5–8 cultures valid for complex network analysis (see Materials and Methods). However, results were comparable when all cultures were used. Numbers of nodes and links followed a comparable trend for two different persistence thresholds (see Figure S1), indicating their robustness to threshold selection. Edge density followed different trends for the different link persistence thresholds; this was due to small differences in the numbers of nodes at each age, resulting in larger differences in edge density (Figure S1).

#### Complex topological properties

**Figure 3:**
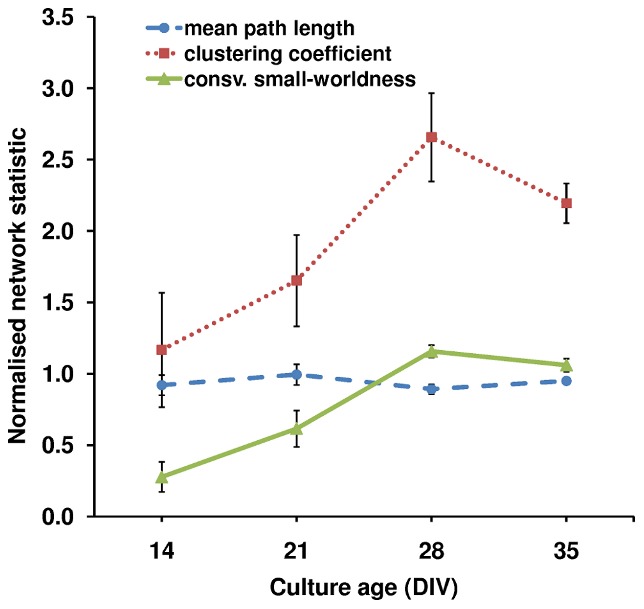
Complex topological properties of the persistent networks as a function of culture age. Mean path length, clustering coefficient and conservative small-worldness; averages (*n* = 5–6), were normalized as follows: mean path length (*L*) and clustering coefficient (*C*) were normalized against the expected value from an equivalent population of random networks (*n* = 50) with the same number of nodes and links. Small worldness was calculated conservatively as (*C*_real_/*C*_lattice_)/(*L*_real_/*L*_rand_). Error bars represent ± s.e.m. The average shortest path length and clustering coefficient at DIV 14 were both close to the value expected for a random network. A statistically significant increase in the clustering coefficient was found between DIV 14 and DIV 28. The combination of a short mean path length and high clustering at DIVs 28 and 35 lead to a network classified as ‘small-world’.

Complex network statistics from each culture's persistent network were used to assess changes in network topology as the cultures matured. Figure 3 shows the progression of network-wide statistics as a function of age: there was a significant (P = 0.018) increase in the mean clustering coefficient between DIVs 14 and 28, whilst mean path length was relatively stable across ages (P = 0.6). The combination of increased clustering coefficient and stable mean path length resulted in a significant increase in the small-worldness property (P≤0.001) and networks were classified as small-world at DIVs 28 and 35. Homogeneous subsets were DIV 14 and 21, and DIV 28 and 35, indicating a change in the small-worldness between the third and fourth week *in vitro*.

**Figure 4:**
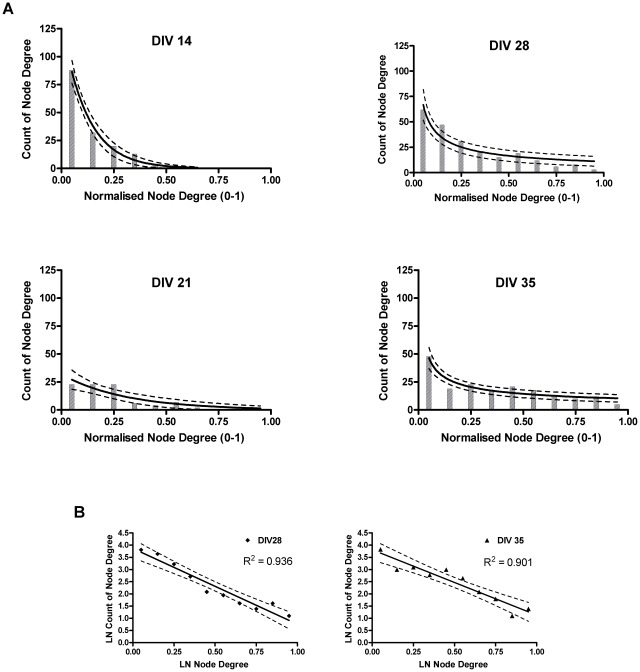
Change in the node degree distribution with culture development. Node degree distributions, obtained from all the nodes of the persistent networks of all cultures using a bin size of 10%. Panel A: bar graphs represent node degree distributions on a linear scale. Solid lines show the best fitting model at each age, broken lines represent 95^th^ percent confidence interval. Top left: DIV 14, bottom left: DIV 21, top right DIV 28, bottom right: DIV 35. DIVs 14 and 21 show exponential fit on a linear scale, DIVs 28 and 35 show power law fit on a linear scale. Panel B: scatter plots represent node degree distributions on a log-log scale, DIVs 28 and 35 are shown with a linear fit. The fat tailed node degree distribution found at DIVs 28 and 35 is indicative of nodes with a high degree (hubs).

To assess the relative influence of nodes in the network, the form of the node degree distributions were compared between ages. As the cultures matured, the number of nodes with a high degree increased, leading to a fatter tailed node degree distribution (Figure 4). To quantify this change, both slow decaying (power law) and fast decaying (exponential) statistical models were fitted to the data (see Materials and Methods). There was a significant increase in the goodness of fit ratio (power law/exponential) as the cultures aged (P = 0.024). The few data points at DIV 14 meant that goodness of fit could not be reliably distinguished between models. However, at DIV 21 the ratio was <1 indicating a closer fit by an exponential model, whilst at DIV 35 the ratio was >1 indicating a closer fit by a power law model. *Post hoc* tests showed that the DIV 21 ratio was significantly smaller than the DIV 35 ratio (P = 0.017, P = 0.021, Tukey HSD and Bonferroni *post hoc* tests respectively).

#### Spatial network properties

**Figure 5:**
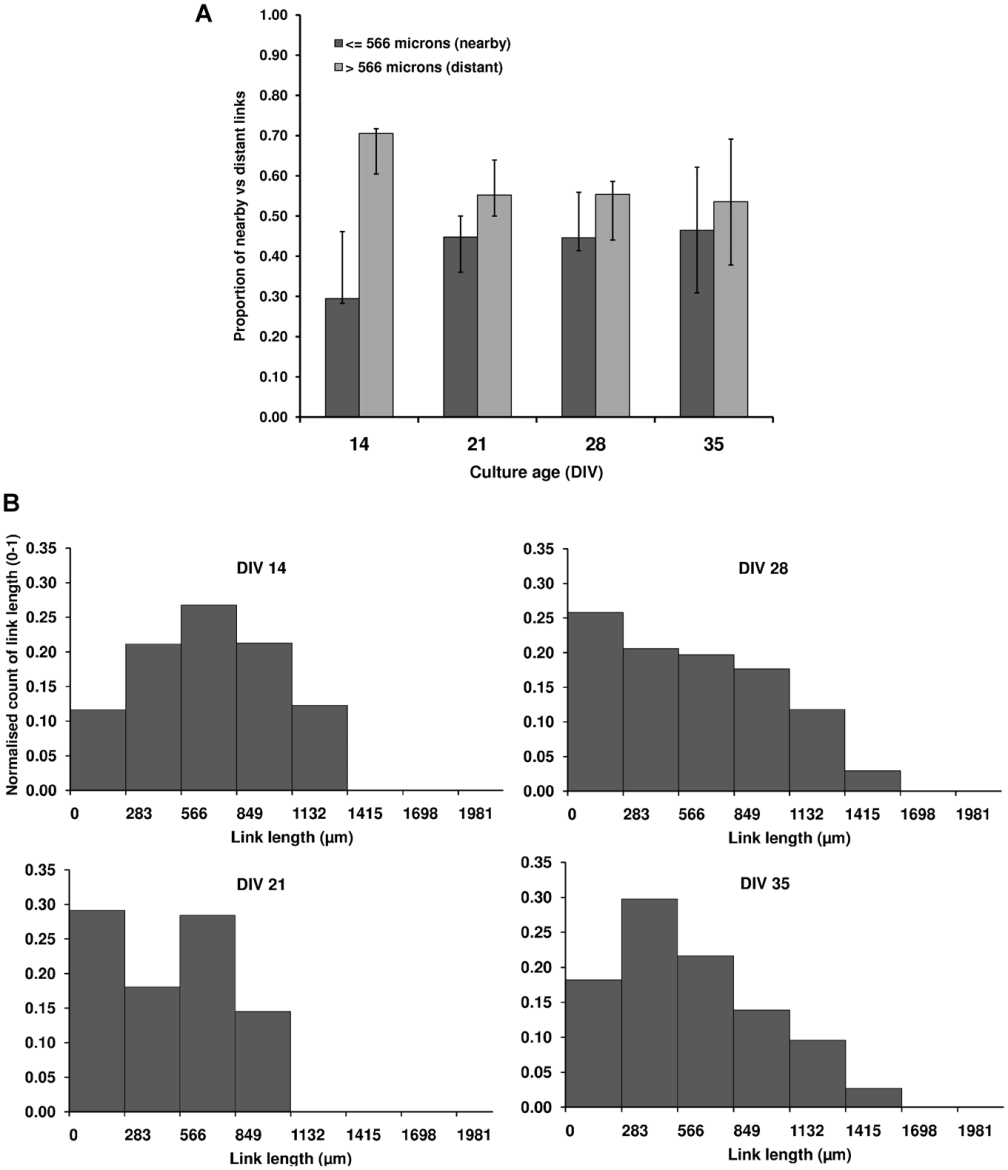
Change in the link lengths with culture development. Panel A: Each bar represents the median proportion of links between nodes up to (and including) two electrodes apart (classified as ‘nearby’) and links between nodes greater than two electrodes apart (classified as ‘distant’), diagonal neighbors were included; values were calculated from all cultures at each age. Upwards error bars represent the 75^th^ percentile and downwards bars the 25^th^ percentile. Notably, at DIV 14 there was a significantly higher number of connections between distant nodes. Panel B: Normalized histograms of link lengths at each culture age, constructed from the link lengths of all cultures, measured as the proportion of each culture's links at each length. Median values from all cultures were used for each bin in the histogram. Bin size was based on spacing between electrodes of MEA, with one bin for each electrode distance (i.e. bin 1 is all links between neighboring electrodes - including diagonal neighbors, bin 2 is all links between nodes up to two electrodes distance, and so forth until seven electrodes distance which is the maximum between any two nodes on the MEA). Bin edges (X-axis) specify the start of each bin, measured as the distance between electrodes on the MEA (micrometers). Y-axis is the same for all histograms in panel, only DIV 14 Y-axis is labeled to avoid overcrowding.

The spatial organization of nodes and links also changed as cultures matured. At DIV 14, the proportion of links between distant nodes was significantly higher than the proportion of links between nearby nodes (P = 0.028), whilst at subsequent ages there was no significant difference (P = 0.27, 0.83, 0.5, for DIV 21, 28, and 35, respectively), Figure 5 panel A. The distribution of link lengths (Figure 5, panel B) is characteristically Gaussian at DIV 14, but appeared bimodal at DIV 21. Notably at DIVs 28 and 35, the distribution was slightly longer tailed and positively skewed (skewness = 0.000, 0.085, 0.352 and 0.572, at DIVs 14, 21, 28 and 35, respectively). Skewness followed a linearly increasing trend between DIVs 14 and 35 (R^2^ = 0.964), reaching significance at DIV 35 (P = 0.018).

**Figure 6:**
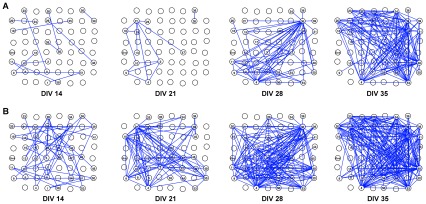
The persistent network of a representative culture at DIV 14, 21, 28 and 35. Graphs illustrate the spatial organization of network components at each culture age: the 8 by 8 grid corresponds to positions of the electrodes on the multi-electrode array (MEA). Nodes that are part of the network (i.e. for which a link was identified) are numbered according to their MEA hardware numbers, and the lines between electrodes represent un-directed links between nodes. Panel A: graphs from the networks thresholded at 25% link persistence. Panel B: graphs from the networks thresholded at 15% link persistence, this lower threshold results in more nodes and links.

Network graphs were generated to depict the spatial arrangement of each culture's network components. Figure 6, panel A shows the persistent network of a representative culture at DIVs 14, 21, 28 and 35. At DIV 14, the graph was a sparse collection of links between often distant nodes. In some cases regions were disconnected from the main graph (as can happen when high thresholds are applied to correlation matrices [8]). At DIV 21, there were fewer nodes and links in some (but not all) cultures. From this age onwards, there was a more even distribution of links between nearby *vs* distant nodes. At DIVs 28 and 35 some cultures had more nodes and links, and there was a trend towards an increase in the number of links between DIVs 14/21 and DIVs 28/35 (see Figure 2). Figure 6, panel B shows the persistent networks from the same culture at a lower link persistence threshold. As expected, there were more nodes and links at each age, nonetheless changes in the numbers of nodes and links followed a comparable trend to the main results (see also Figure S1).

**Figure 7:**
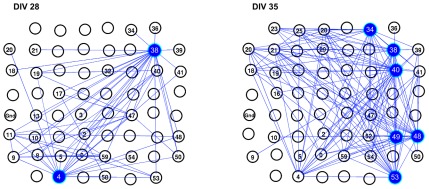
Visualization of hubs in a representative culture at DIVs 28 and 35. Graphs illustrate the location of hubs in the persistent network of a representative culture at two separate ages. The 8 by 8 grid corresponds to positions of the electrodes on the multi-electrode array (MEA). Nodes that are part of the network (i.e. for which a link was identified) are numbered according to their MEA hardware numbers, and the lines between electrodes represent un-directed links between nodes. At DIV 28 (left hand graph), nodes 4 and 38 were classified as hubs in the network, whilst at DIV 35 (right hand graph), nodes 34, 38, 40, 48, 49 and 53 were hubs. Hubs were classified as nodes having a high degree (degree greater than mean node degree plus one standard deviation) and are highlighted with blue circles.

Figure 7 shows a close up of one culture's network at DIVs 28 and 35, highlighting the position of high degree nodes (hubs) [18] in the networks. Since cultures have no pre-built infrastructure, and the cells are randomly distributed over the MEA, the absolute position of the hubs in the culture dish is not of particular interest. However, the relative position of the hubs (with respect to the nodes that they connected to) may reveal patterns such as whether hubs are located in close proximity to one another, or have a higher proportion of links to distant *vs* nearby nodes. There are numerous potential patterns and it was not possible to evaluate them for the present study. However, the figure is intended to illustrate some of the possibilities for future research.

### Network properties and activity propagation

Results presented thus far have focused on identifying changes in the network infrastructure (via the persistent interactions between different areas [nodes] in the cultures). Here, the results focus upon the activity that takes place over this infrastructure. Each transient network is considered as a ‘snapshot’ of network activity, measured over a short time-scale (duration of a network-wide burst) and reflects interactions between different areas of the culture in this period.

**Figure 8:**
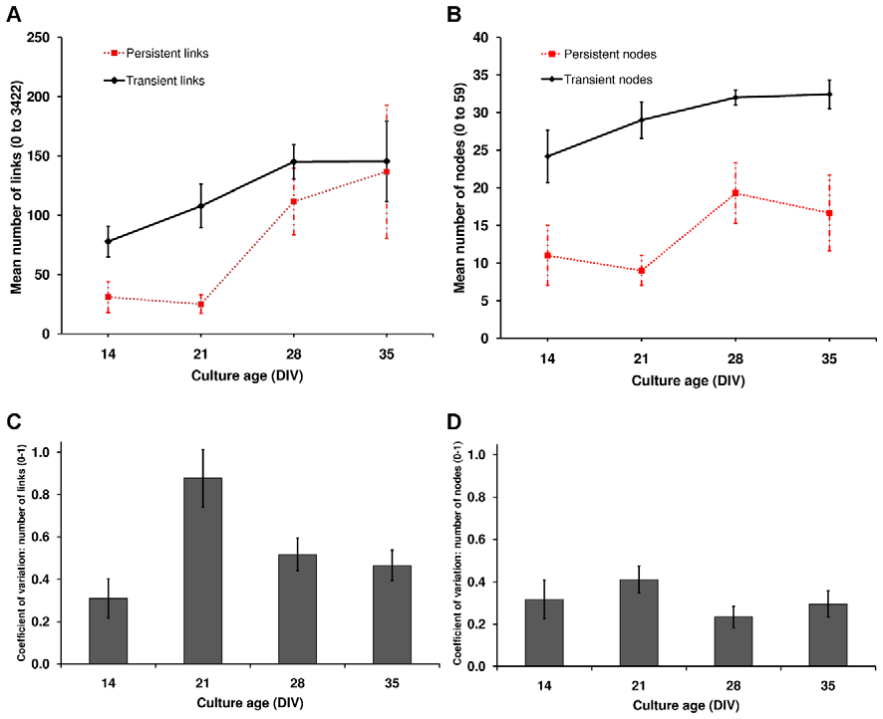
Basic topological properties of the transient networks as a function of culture age. Panels A, B: mean number of links and nodes (respectively) in transient networks, averaged over all cultures at a particular age (solid black lines). Error bars represent ± s.e.m. The mean numbers of links at each culture age suggested an increasing trend in the number of links between DIVs 14 and 28, however the trend was not significant (P = 0.087). Likewise the mean numbers of nodes suggested an increasing trend (P = 0.089). The mean numbers of persistent network links and nodes are shown for reference (dotted red lines). Panels C, D: expected coefficient of variation for the numbers of links and nodes (respectively) in each culture's set of transient networks. Error bars represent ± s.e.m. Coefficient of variation for number of links was significantly higher at DIV 21 than DIV 14 (P = 0.021).

As per the persistent networks, the basic properties relating to network size were compared. Additionally, since there were multiple transient networks for each culture, the coefficient of variation was also analyzed (see Materials and Methods). Figure 8 panel A shows the expected number of transient links as a function of culture age, panel B shows the equivalent data for number of nodes. There was a strong trend towards an increase in the mean number of transient network links (P = 0.087), and a strong trend towards an increase in the number of nodes (P = 0.089). Figure 8 panel C shows the expected coefficient of variation for the number of transient links. This was largest at DIV 21 and there was a significant increase in coefficient of variation between DIV 14 and DIV 21 (P = 0.021). This demonstrated that transient networks at DIV 21 varied considerably in their numbers of links, more so than at any other age. Panel D shows the equivalent data for number of nodes (no significant difference).

#### Influence of functional network properties on efficiency of activity propagation

**Figure 9:**
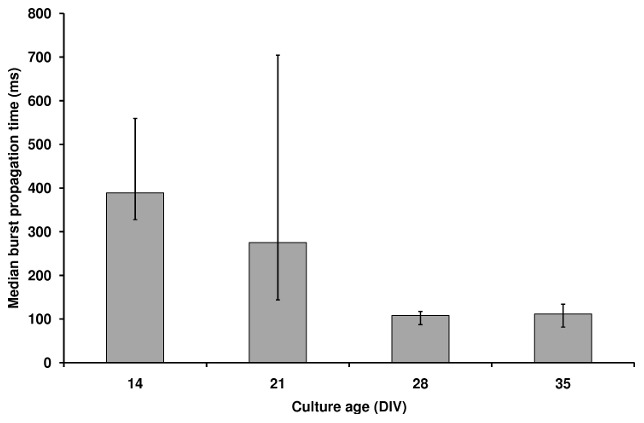
Network-wide burst propagation time as a function of culture age. Bar chart shows the median burst propagation time (from all transient networks of all cultures at each age), values outside the 5^th^ to 95^th^ percentiles were removed as outliers, giving *n* = 6–8 for each age). Error bars show 25^th^ and 75^th^ percentiles. A (network-wide) burst was defined as a near-simultaneous (within 250 ms) occurrence of channel bursts on multiple (≥4) channels. A channel was considered to display bursting activity if ≥4 spikes were detected in 100 ms. For each channel included in the burst, recruitment time was the timestamp of the first spike in the ≥4 spikes in 100 ms sequence. Burst propagation time was calculated as the time to recruit all channels in a network-wide burst. At DIVs 28 and 35, this time was significantly lower than at DIV 14.

To assess whether network properties influenced the transfer of information across the culture, burst propagation time was compared at each age (Figure 9). There was a significant difference in the median burst propagation times (P = 0.002), with DIV 14 significantly different to DIVs 28 and 35 (P<0.05). At DIV 14, median burst propagation time was highest (389 ms), and although it reduced to 275 ms at DIV 21, variability was highest at this age. Burst propagation time further decreased between the remaining ages (to 108 ms at DIV 28, and 112 ms at DIV 35). Variability of the burst propagation times showed a large reduction between DIV 21 and DIV 28 (inter quartile ranges 577 ms, 36 ms, respectively).

#### Influence of functional network properties on reliability of activity propagation

**Figure 10:**
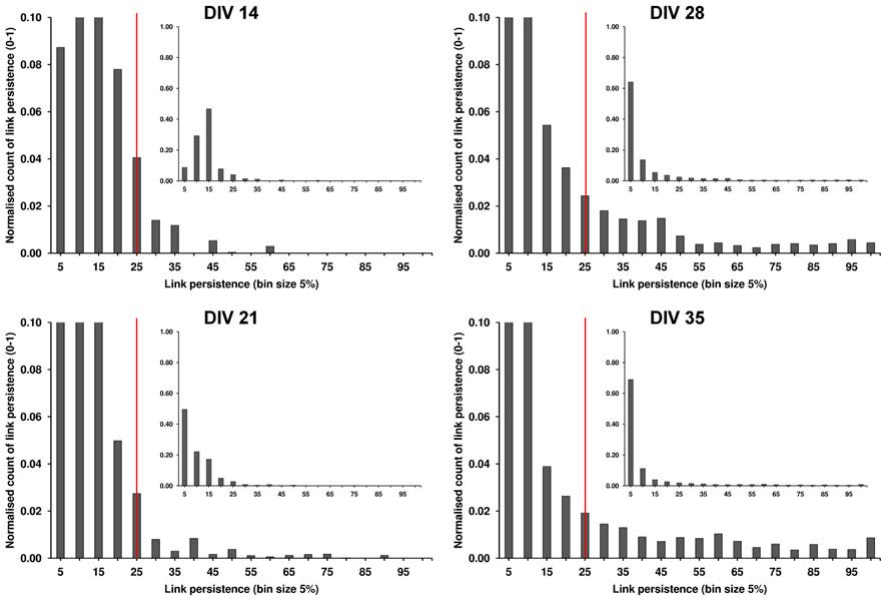
An increase in the number of links with high persistence as cultures aged. Each histogram shows the percentage of links found at each link persistence level for all cultures at each age (normalized count of links found at the persistence value, expressed as the percentage of transient networks (bursts) in which the link was found, bin size 5%, bin edges specify the end of each bin). Top left: DIV 14, bottom left: DIV 21, top right: DIV 28, bottom right: DIV 35. Red (solid) line is the link persistence threshold (link presence in at least 25% of the transient networks). The histograms are cropped to show the detailed distribution, inset histograms show the full scale. At DIV 21, many links were found infrequently (i.e. below the link persistence threshold). The more pronounced tail of the distribution as the cultures matured, reflected a significantly higher contribution of persistent links in the network's of mature cultures.

To investigate whether links became more reliable (persistent) as the cultures matured a histogram of link persistence values was generated. The fat tailed link persistence distributions at DIVs 28 and 35 reflected the fact that persistent links became more numerous and were activated more frequently (Figure 10). There was a significant increase in the contribution of the persistent links as the cultures aged, P = 0.044, (mean ranks: 1.50, 1.75, 3.00, 3.75). On closer inspection, the increase was for the contribution of links in the 50 to 75% link persistence categories (P = 0.010).

## Discussion

The present study characterizes the evolution of functional networks observed in cortical cultures and extends previous work where network properties of cultures were investigated at a single developmental stage [34], [39]. Analysis of activity from multiple bursts allowed the identification of frequently activated links - the persistent network, which was robust to inter-burst fluctuations in activity and suitable for analysis of complex network statistics. Results demonstrated that cortical cultures exhibit developmentally-dependent structured interactions, which are reflected in their persistent patterns of activity. These data suggest the evolution of a complex network of links that supports increasingly efficient information flow and specialized processing. Given the absence of external chemical or electrical stimulation applied to the cultures, these findings support the assertion that such complex network evolution is an intrinsic property of neuronal maturation. Moreover, the characterization of age-dependent network properties enables appropriate selection of culture development stages for specific experiments [24], [37], [38], [42].

### Unstructured interactions in the spontaneous activity of immature cultures

Immature cultures (DIV 14) exhibited limited interactions between neuronal units, resulting in a network of few nodes and links. The observation that at DIV 14 activity could spread rapidly between any two neuronal units (short mean path length in Figure 3, reflects high integration), but was slow to propagate network-wide (Figure 9) indicated an absence of functional organization. The homogeneous node degree distribution and low clustering coefficient exemplified the poor functional differentiation between nodes, with no evidence of densely interconnected areas that could support segregation of neural processing. Together, these network properties implied a disordered spread of activity, across a random network topology, whilst the long burst propagation time indicated an inefficient structure for widespread information transfer. Since dissociated neurons were seeded randomly onto the MEA and received no external stimulation, it could be expected that their initial connectivity resulted in a random topology. Moreover, since neuron-synapse maturation is incomplete at DIV 14 [24], [53], it is unsurprising that the complex network properties found in mature cultures [39] were not present at this age. However, the prevalence of long-distance connections at DIV 14 (Figure 5 and [36]) is counter to the economy of wiring principle [54] and suggests that units are not simply making spatially convenient connections. In *in vivo* and *ex vivo* preparations the cell type and neurochemical identity have been proposed as guiding influences for connectivity [55] and there is evidence that the variety and proportions of neuron types in cortical cultures are similar to those found *in vivo* [25], [27], [56], therefore connectivity in cultures could be similarly guided by these influences.

### Development of a small-world network during culture maturation

Whilst interactions at DIV 14 were clearly unstructured, the subsequent 14 days of development represented a critical window, during which functional complexity increased (Figure 3), leading to the emergence of the small-world topology at DIVs 28 and 35. Figures S2, S3 and S4 demonstrate the robustness of the small-world result.

We consider the possible driving forces behind this topology change to include the level of synchronization, the ratio of excitation-inhibition and the mechanism of Hebbian learning.

Synchronization of culture activity can be defined over a range of timescales – from ‘synchronous busting’ [57], where areas of the network are synchronously *active* (usually to within ∼100 milliseconds), to precise synchronization between the spike times of two or more neural units [36] (usually to within ∼10 ms or less). For the present study, the network links were derived from firing-pattern correlations and thus represent synchronization levels between neural units (nodes); the low number of nodes and links at DIV 14 reflects a low level of synchronization (i.e. between only a few units), compared to a high level of synchronization (i.e. between many units) at DIVs 28 and 35. Literature indicates that a low level of synchronization at DIV 14 may be due to an excitatory-inhibitory imbalance [53]. Conversely, evidence suggests that a high level of network-synchronization found in older cultures (whereby many neural units are activated within a short time-window [24]) is supported by a balanced excitatory-inhibitory subsystem [53], with tight synchronization between pairs of neural units (as observed in [36]) arising from the activity-based refinement of synaptic connection strengths [24], [58].

In a previous study of functional connectivity during development [36] culture properties at DIV 14 and DIVs 28–35 are in accordance with those of the present study. However, at DIV 21 [36] reported an increased level of synchronization and a dramatic change in burst properties (compared to those at DIV 14). In contrast, the present study revealed no such increase in synchronization at DIV 21, yet burst properties were highly variable - as reflected by a highly variable number of transient links (Figures 2,8), and there was a highly variable burst propagation time (Figure 9). Results herein suggest a network with an uneven balance between highly and poorly interconnected areas, whereby bursts initiated from different sites (as reported in [59]) propagate at different rates, with little link activation regularity (as reflected by the low link persistence at this age). We posit that the highly variable burst properties reported herein and in [24], [36] point to itinerant rather than persistent synchronization at DIV 21. Such transient synchronization effects may be averaged out by requiring multiple occurrences of correlated activity over long time-scales [58]. Therefore, our persistent networks at this age may not reflect the increased synchronization found in [36] (where links required only a period of correlated activity during the entire recording).

Crucially, the combination of varied burst properties and transient synchronization at DIV 21 indicates a mixture of regular and irregular activity. Modeling studies have suggested that such mixed activity constitutes optimal conditions for the emergence of a small-world topology via Hebbian learning rules and activity driven plasticity [60]. Thus, a change in the culture's spontaneous activity patterns could drive the topology transformation. Results herein and in [61] suggest that once the topology of the network has emerged, equilibrium states may exist at different time scales - from transient synchronization between subgroups of neural units at the short time-scale to regular occurrence of such transiently activated subgroups over longer time-scales. Modeling studies may provide further insight into the role of synchronization and the evolution of such equilibrium states [62], whilst pharmacological manipulation of specific neuron sub-types could verify biological mechanisms behind activity modulation.

#### Increased clustering of connections

Our results demonstrate that functional clustering increases from DIV 14. Moreover, this increased clustering (rather than a reduced mean path length) was the cause of increased small-worldness, which continued until cultures reached a state of semi-maturity at DIV 28. We note that the increased clustering was accompanied by a change in the distribution of link lengths, from a clear dominance of long-range links at DIV 14 to an increased proportion of short links thereafter, which suggests an increase in localized lattice-like clustering. The change of the link length distributions from Gaussian to bimodal, to long-tailed at DIVs 14, 21 and 28–35 respectively (Figure 5 panel B), coincides with the network topology shift from random, to mixed, to small-world. Moreover, the small proportion of long-range links at DIVs 28 and 35 suggests connections between distant areas – perhaps between clusters. Together, these findings suggest that spatial considerations may also play a role in the topology change.

#### Increasing presence of hubs

During culture maturation, the distribution of node connections changed from a rapidly decaying and homogeneous degree distribution to one with a longer tail, indicating a small but non-negligible proportion of highly connected nodes (hubs). The small-world topology does not require hubs [51] and both random and lattice networks have a single-scale degree distribution. Nonetheless, hubs have been identified in various small-world networks [9], [18], [35]. Moreover, modeling studies imply that presence of a non-Gaussian degree distribution is more likely when networks of neurons evolve from irregular firing [60], thus it is plausible that the irregular burst properties at DIV 21 may be related to the formation of hubs.

### Mature cultures and the influence of network topology on activity

Networks at DIVs 28 and 35 were classified as small-world, exhibiting several highly connected areas (clusters of highly inter-connected neural units), alongside the ability for any two areas to interact via few intermediary connections (short mean path length). Interestingly, when the network properties at DIVs 28 and 35 were compared, smaller differences were found than between earlier ages, suggesting a state of maturity [24], [32], [36], [63]. The non-trivial network structure demonstrated at DIVs 28 and 35 corresponds well with previous work [39], which concluded that mature cultures had complex network properties similar to those found *in vivo*.

Small-world networks have an architecture which supports efficient information transfer [8], [64]. Accordingly, our results showed a developmental reduction in burst propagation time that accompanied the emergence of cultures' small-world properties (Figure 9). Furthermore, variability of burst propagation time was lower at DIVs 28 and 35 than at younger ages. Since burst events are typically initiated from a number of sites [59], this reduced variability suggests that burst propagation times in mature cultures are not influenced by burst source; information propagates efficiently from all parts of the network. Interestingly, a small-proportion of links at DIVs 28 and 35 were activated extremely frequently (Figure 10), suggesting that they facilitate many of the interactions; it is possible that they represent activation of the small-world ‘short cuts’ between clusters.

The increasing prevalence of highly connected nodes in older cultures suggests that such hubs play a greater role in network activity as the cultures mature, perhaps indicating sources [65], sinks, or bridges [18], [33] for network activity. Interestingly, structural and functional hubs have recently been identified in the developing hippocampus where GABAergic interneuron hubs were found to orchestrate network synchrony [52], firing immediately prior to network bursts. Similarities between connectivity of GABAergic interneurons in the hippocampus and neocortex [66] and suggestions that cortical cultures develop subsystems akin to those found *in vivo* [25], [53], [56], imply that similar functional hubs may be present in the primary cortical cultures employed herein.

### Conclusions and future work

The present study has demonstrated that networks derived from the spontaneous activity of cultures develop non-random properties despite a lack of external input. Based on these results, we draw four main conclusions. Firstly, to mitigate fluctuations in spontaneous activity, multiple network bursts should be assessed to obtain the persistent network. Secondly, the functional network of a cortical culture evolves from an initial random topology to a small-world topology; we propose this is due to a change in the culture's spontaneous activity patterns that is driven by the maturing excitatory-inhibitory balance and an increase in network-wide synchronization. Thirdly, the reduction in burst propagation time with culture maturation that accompanies the evolution of a small-world topology supports the efficient network-wide flow of information afforded by a small-world network. Lastly, the presence of hubs and increasing contribution of links with high persistence suggests a proportion of highly influential nodes and links.

To the authors' knowledge, this is the first demonstration of small-world properties evolving in the functional networks of cortical neurons grown *in vitro*. This further supports work suggesting maturation of *in vitro* networks around the age of DIV 28 to 35; importantly, our results indicate that experiments which require complex network features should be undertaken from DIV 28 onwards, whilst those aiming to shape network maturation should be undertaken before DIV 28. Moreover, the work herein further supports the use of complex network statistics to quantify network level changes resulting from different experimental conditions, and importantly it provides a benchmark against which to assess the influence of closed loop stimulation on shaping cultures network properties - a fundamental question for the work on closed loop culture embodiment.

An important area for future work is to investigate the role of frequently activated nodes (hubs) in cultured neurons; including whether the presence of network-synchrony controlling hubs in the underlying substrate could mediate the timing and extent of functional interactions between otherwise segregated clusters, perhaps coordinating synchronous network-wide bursting. Additionally, the use of staining to identify the location and proportion of the different neuron types and sub-types, and the use of pharmacological manipulation to verify their effect on activity may help elucidate mechanisms behind the different network properties.

## Materials and Methods

### Cell cultures and sample population selection

Data used for the present study was collected for [44], from cultures of pre-natal (E18) rat dissociated cortical neurons and glia cells, seeded onto multi-electrode arrays (MEAs, Multi Channel Systems, Reutlingen, Germany). Cultures were maintained in Teflon sealed MEAs in an incubator at 5% CO_2_, 9% O_2_, 35°C and 65% relative humidity [44]. For the present study, ‘dense’ cultures (estimated cell density of 2,500±1,500 per mm^2^) were used.

Culture's electrical activity was recorded daily during their first 5 weeks of development. For the present study, a sample population was selected from the large number of cultures recorded, specifically, 10 cultures from 4 preparations (plating batches). Cultures were arbitrarily selected from those that had recordings every 7 DIV, i.e. those which survived for the full 5 weeks and for whom none of the weekly recordings were missed. The use of multiple preparations is important as bursting patterns across preparations vary considerably [44]. Additionally, since the variation in burst properties measured at the same age (DIV) from different cultures (of the same plating), can exceed day-to-day differences in their properties (and inter-plating differences are significantly larger) [44], network properties were compared at weekly intervals. This also allows easy comparison with results from other studies [32], [36].

### Electrophysiological recording

Data were recorded from cultures for 30 minutes daily in the incubator used for culture's maintenance. Unit and multi-unit spontaneous spike firing was recorded from the MEA (8×8 array of 59 planar electrodes, each 30 µm diameter with 200 µm inter-electrode spacing [centre to centre]). The pre-amplifier was from Multi Channel Systems (MCS), excess heat was removed using a custom Peltier-cooled platform. Data acquisition and online spike detection was performed using MEABench [67]. According to the MEA user manual (MCS) spike detection is reliable up to ∼100 µm from the electrode centre, beyond which spikes become indistinguishable from the background noise. Therefore, each MEA provides a grid of 59 non-overlapping 100 µm recording horizons (once the four analogue channels and single ground electrode are removed). It should be noted that data recorded on each channel may be from multi-neuron activity, no attempt was made to spike sort the data as overlapping waveforms found during a burst can present problems [44]. Lastly, as recording began immediately after the cultures were transferred to the pre-amplifier, the first 10 minutes were discarded from the analysis in order to mitigate any movement induced changes in culture activity [44], [68].

### Data pre-processing and burst detection

Spikes were detected online (using MEABench), positive or negative excursions beyond a threshold of 4.5× estimated RMS noise, were classed as spikes. Their peak amplitude timestamp (µs), plus electrode number were stored. For the present study, all positive amplitude spikes were removed to avoid counting spikes on both upwards and downwards phases.

In cortical cultures, global bursts (population bursts), characterized by an increase in culture activity across the entire MEA, are typically present from DIV 4–6 onwards [44], but sometimes as late as DIV 14 onwards [36]. Such bursts provide a time window during which many culture interactions take place and thus a useful opportunity to assess network-wide connectivity. For the present study, global bursts were identified as an increase in the number of spikes detected per unit time, summed over all electrodes in the array: specifically ≥4 spikes per channel in 100 ms, on ≥4 channels within 250 ms; based on the SIMMUX algorithm, included as Matlab (The MathWorks, Natick, MA, USA) code with MEABench. Burst start was determined by the timestamp of the first spike included in the global burst, and burst end taken as the timestamp of the last spike included. To assess interactions between neural units underlying all the electrodes, global bursts in which at least 25% (15/59) electrodes registered channel bursts (≥4 spikes in 100 ms) were selected. These were termed ‘network-wide’ bursts and ensured that many neural units participated in the burst (increasing the probability that the resultant networks would have sufficient numbers of nodes for the analysis of network properties). Additionally, since there were typically 10 to 150 such bursts in the 20 minute recording segment used, it provided a good balance between having sufficient numbers of bursts for analysis, whilst avoiding the inclusion of ‘tiny’ bursts [44] since these may have biased results.

All activity occurring from the first spike in the nw-burst to the last spike in the nw-burst (including tonic activity from electrodes not included in the nw-burst) was used for assessing the relationships between channel pairs. Spike occurrences were counted in 1 ms bins, this allowed a certain amount of jitter in the spike arrival times (which could otherwise decrease the likelihood of identifying correlated activity). Bin size was selected based on experimentation with 1, 5 and 10 ms bins. The 1 ms bins provided a greater separation between correlated and un-correlated channels, data not shown.

### Link definition: Cross covariance

Functional connectivity was assessed by correlating spike times recorded on pairs of electrodes during a network-wide burst (as per [34]). This linear link analysis method assesses the probability of a spike at time *t* on one electrode being accompanied by a spike arriving at *t*±*k* on another electrode, where *k* is the allowable lag time. Spike times arriving within ±13 ms of each other were considered to be related (under the assumption that a linear relationship between spike arrival times on pairs of electrodes indicates their coupling). The maximum lag time was based on speed of axonal propagation, time for synaptic transmission and the maximum distance between 2 points on the MEA. Since the firing rates recorded on each channel may be different, cross-covariance was used, this correlates deviations in firing rates from their respective means as a function of lag.

Channels that had fewer than 8 spikes recorded during the burst were excluded from the cross-covariance analysis, as results from synthetic data testing showed that performing cross-covariance on vectors with fewer than 8 spikes was poor at distinguishing related vectors from independent ones (data not shown).

The cross-covariance function calculates the covariance of two random vectors:
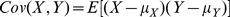
(1)In the case where *X* and *Y* are time-series the cross-covariance may depend on the time when it is estimated and on the lag between the time series:
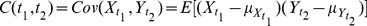
(2)For wide-sense stationary time series, covariance is a function of the lag only:
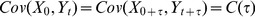
(3)Cross covariance was calculated using the built in Matlab function xcov; specifically, each pair of channels with at least 1 ms overlap in their activity were compared from the time of the first spike on either channel to the time of the last spike on either channel. The tightness of the correlation window (*X* or *Y* channel recording spikes), and requirement for overlapping activity was to mitigate the effects of long periods of quiescence and to ensure that the data were as wide-sense stationary as possible.

Calculation of the cross-covariance at each lag resulted in a cross-covariance plot for each channel pair. The maximum cross-covariance value (peak of the plot) was used to determine whether a link between nodes was present by comparing it to a threshold as detailed next.

#### Transient network link definition threshold

Under the assumption that a peak in the cross-covariance (XCov) plot indicates a relationship between the channel pairs [34], the link definition threshold was set at 4 times the expected value of uniformly distributed cross-covariance bin counts (the sum of the counts from all 26 bins excluding 0 lag, divided by the number of bins). It was decided not to use a fixed threshold, since the mean XCov value increased as the cultures matured. Moreover, it varied between cultures and the age related increase did not occur in the same way for each culture. Thus, when a fixed threshold was used a proportion of the networks obtained were either too small/sparse, or too dense for analysis of their complex network properties (empirical). By setting a threshold that identified peaks in the plot, the results were not influenced by variations in the mean cross covariance level. Figure S5 shows the ability of the threshold to identify true positives and reject false positives. The threshold calculated for each channel pair was applied to the weighted adjacency matrix, providing a binary adjacency matrix (transient network) for each nw-burst. The matrices were symmetrized (i.e. if a link was found in one direction, a corresponding link was added in the opposite direction).

The transient networks obtained over the duration of a recording were found to be highly variable (see Results), therefore to obtain a more robust estimate of the network infrastructure, a persistent network was calculated as the set of most frequently activated links over all transient networks.

#### Persistent network

To compute the persistent network a weighted adjacency matrix comprising the count of each link's occurrence over all transient networks was obtained by summing the binary adjacency matrices of all transient networks. A link persistence threshold was applied to this ‘adjacency frequency matrix’ to obtain a binary adjacency matrix representing the persistent network. At a threshold of 1, the persistent network is simply the superset of all transient networks (and thus, not strictly speaking, ‘persistent’); conversely a threshold set equal to the total number of transient networks, requires link presence in every transient network. Setting the threshold equal to link presence in 25% of transient networks provided a good balance between minimizing the number of overly dense and overly sparse networks (see complex network analysis).

### Complex network analysis: Topological properties

Table S1 provides the mathematical definitions for the topological properties and complex network statistics. Basic topological properties (related to network size), and complex network statistics, were calculated from the adjacency matrices (using Matlab, with additional scripts from the Brain Connectivity Toolbox [14]). For each transient network, only basic topological properties were measured, complex network statistics were not calculated due to the highly variable network size and edge density (see verification of network size and edge density). Instead, the mean numbers of nodes and links were calculated over all transient networks in the recording. Additionally, the coefficient of variation for number nodes and for number of links was calculated over all transient networks in the recording. The expected numbers of nodes, and links and the expected coefficients of variation were calculated over all 10 cultures.

#### Verification of network size and edge density for complex network analysis

Since some of the complex network statistics are defined only for certain ranges of network size, it was important to ensure that each persistent network was within the size range suitable for complex network analysis. Specifically, an assumption made when assessing small-world properties is that the networks are sparse: *n*>>*K* [16] (where *n* is number of nodes and *K* is mean node degree). Since the maximum *K* is constrained by the number of nodes, this verifies that the average number of connections per node is much lower than the total number of nodes in the network (i.e. the graph is far from being fully connected and is thus ‘low-cost’). To check for the required sparseness, an edge density (cost; number of links/number of possible links) in the range 0.05 to 0.34 was sought (following [8]). To ensure that sufficient numbers of networks met the criterion at each age, whilst avoiding too many overly sparse networks (those with *K*<log(*n*)) [16], three different persistent link definition thresholds were tested: 0.15, 0.25, 0.35. These resulted in 3 sets of networks with increasing numbers of nodes and links (and a variety of edge densities). The selected threshold (0.25) provided the best balance between minimizing the number of overly dense and overly sparse networks, and provided a set of networks where 83% had an edge density in the range 0.05 to 0.34. Additionally, this threshold resulted in the least variation of edge density between ages; this was useful for comparing statistics influenced by edge density, such as clustering coefficient.

#### Calculation of network statistics

The expected persistent network statistics for each age (DIV) were obtained from all 10 cultures. For the numbers of nodes, links and edge density, data outside the 5^th^ to 95^th^ percentile were removed as outliers (maximum removed = data from 4 cultures, leaving minimum *n* = 5 at all times, once cultures failing to meet the edge density criterion had been removed). The power of statistical tests was verified to ensure that *n* numbers for each network statistic were sufficient (see Significance testing subsection).

For each persistent network, the network-wide statistics (mean path length [average shortest path length] (*L*), global efficiency (*E*), mean clustering coefficient (*C*), small-worldness (*S^ws^*), and mean node degree (*K*)) were calculated over all nodes that had at least one link (and in the case of mean path length, over all node-pairs with non-infinite distances [3]). *C*, *L* and *E* were normalized against expected values from a population of equivalent random networks with the same number of nodes and links (see section on generation of equivalent null hypothesis networks).

Small-worldness of the network [17] was calculated using the Watts and Strogatz [16] definition of clustering coefficient, by taking the ratio of normalized mean clustering coefficient to normalized mean path length. Here, clustering coefficient was normalized to the value expected for an equivalent lattice network, and mean path length to the value expected for an equivalent random network, this provided a conservative estimate of small-worldness (see Figure S6).

To check that the small-world metric was not influenced by network disconnectedness (as mean path length is only defined for connected graphs and not all graphs were connected), the ratio of global efficiency to the clustering coefficient [8] was also compared (Figure S4). Global efficiency (*E*) is inversely related to mean path length and is suitable for use on connected or disconnected networks. Thus, replacing mean path length (in the small-world calculation), with 1/*E*, enabled calculation of the small-world metric based on global efficiency.

#### Assessment of node degree distributions

Node degree distribution was calculated using all nodes of the network by counting the number of nodes with each degree in bins of size 2. Bin size was selected to provide a sufficient number of data points, whilst minimizing the number of empty bins (sizes 1, 2 and 3 were tested, data not shown). Hubs were identified as nodes with a degree greater than mean node degree plus one standard deviation [18]. To assess if the node degree distribution followed exponential or power law trend, both of these distributions were fitted to the node degree data using Graph Pad Prism 4 (GraphPad Software, Inc., La Jolla, CA, USA). The goodness of fit ratio of power law to exponential model was calculated for each culture at each age (to test the null hypothesis that data would not differ from an exponential fit). The degree distributions P(*k*) were of the following form: exponential, P*(K)*∼*e* ^−*αK*^; and power law, P*(K)* = *k* ^−*α*^.

#### Generation of equivalent null hypothesis networks

Network size and density may influence the magnitude of complex network statistics [69]. To counter this, empirical network properties were compared to both random and lattice null hypothesis networks. Firstly, as per other studies the significance of empirical network statistics was assessed using random networks with the same number of nodes and links to generate a null distribution of the network statistics. Thus for each persistent network (from one culture at a particular age), a set of 50 equivalent random networks was generated (using a script from the Brain Connectivity Toolbox), providing 500 (50×10 cultures) equivalent random networks for each age. As per the real networks, statistics were calculated for the random networks of each culture. The expected random network statistics were then calculated for each culture. Secondly, to assess the significance of the clustering coefficient for a conservative estimate of small-worldness, the expected clustering coefficient from an equivalent lattice network was used. Lastly, comparison of the raw empirical network measurements against those of both equivalent random and lattice networks allowed results to be validated against the upper and lower limits expected (see Figure S3). NOTE: For the alternative link persistence thresholds, the mean path length and clustering coefficient values expected from a population of equivalent random networks were approximated using: *L_Rand_*∼ = ln(*n*)/ln(*K*−1), and *C_Rand_*∼ = *K*/*n* [64].

### Calculation of non-topological properties

In addition to the networks' topological properties, the spatial and temporal features of the networks were also assessed; link distance was calculated as the Euclidean distance between the electrodes on the MEA, based on 200 µm centre-to-centre spacing of the electrodes. For the present study, connections between nodes up to 566 µm (2 electrodes) apart were considered as ‘nearby’ and those greater than 566 µm as ‘distant’. Link persistence was calculated using the weighted persistent network adjacency matrix (i.e. prior to thresholding), normalized so that the persistence value was the percentage of transient networks in which the link was found.

For both link length (derived from the distance between connected nodes) and link persistence, histograms were obtained over all links from all cultures at each age. Thus, for link length, a count of the number of links in each bin (bin size = 1 electrode spacing) was calculated for each network, this was normalized to the total number of links in the network. For link persistence, a count of the links at each persistence level (bin size 5%) was calculated for each network. In both cases, median bin values were obtained over all 10 cultures, therefore the histogram proportions may not always sum to 1.

To quantify the changes in link length and persistence, two further measures were assessed: for link length, the proportion of links between spatially nearby *vs* distant nodes was calculated for each culture, and the median of these values was used to compare results between ages; for link persistence, the contribution of persistent links was measured as the number of links in each 5% persistence category multiplied by the category persistence value (e.g. if 20% of links were found in the 10% persistence category, the contribution was 200). The link contribution counts were further binned into transient (<25%) and persistent (≥25%).

The efficiency of information broadcast was measured as burst propagation time (time to recruit all channels in a network-wide burst). This was calculated in milliseconds from the time of the first spike in the burst, until the time at which all channels participating in the burst had been recruited. Channels could be recruited to the burst whilst the burst was in progress (i.e. sufficient channels displayed the required activity) but once the number of channels bursting dropped below the threshold, channels could no longer be recruited. For each channel included in the burst, recruitment time was the timestamp of the first spike in the burst activity sequence. Burst propagation times were calculated for all bursts of a culture at each age and the median of these was calculated for each age. Outliers (values <5^th^ and >95^th^ percentile) were removed from the data.

#### Visualization of network graphs

Network graphs were visualized using a freely available script [70]. The script was modified to display the node numbers as their corresponding MEA hardware numbers (0 to 59), with Gnd indicating the ground electrode. A further modification was made to highlight the nodes which had high numbers of links (defined as mean number of links plus one standard deviation), these were considered to be ‘hubs’ in the network [2].

### Significance testing

All statistics were obtained using SPSS version 17.0 (SPSS Inc., Chicago, USA). Unless otherwise specified P<0.05 was set as the significance level. Statistical tests for each network property were selected based on the experiment design and form of the resultant data; Checks were performed to ensure that the assumptions of each test were met. Following test selection, statistical power was verified at the 80% level (checking that the proposed test statistic had sufficient power to detect a genuine effect [71] [typically set to a difference of 1–2 times standard deviation of the mean], given the *n* numbers and variability of the data). For the present study, where some of the tests were applied to data with relatively low *n* numbers it was important to ensure that the power of each test was sufficient [72]. It was also important to ensure that the assumptions of the statistical tests were not violated (Text S1 describes the selection and validation of statistical tests used in the present study). The selected tests were as follows:

To check for a significant increasing or decreasing linear trend of the network properties as a function of the culture age, results for each network property were compared using a one-way ANOVA. Culture age (DIV) was the factor, and the network property was the dependent variable. The following properties were assessed in this manner: number of nodes, number of links, edge density, normalized mean path length, normalized clustering coefficient, small-worldness, goodness of fit ratio. In cases where a significant trend was found, Bonferroni and Tukey post-hoc tests were performed to check for significant differences between each pair of conditions, where found, the homogeneous subsets are mentioned in the results. Homogeneity of variances was tested using the Levene test.

Normality was tested using the Shapiro-Wilk normality test. In cases where the sample means were not normally distributed, non-parametric tests were used. For the burst propagation times a Kruskal-Wallis test was performed on the median burst propagation times for each culture at each age, with culture age as the grouping factor and median burst propagation time as the dependent variable. For the proportion of links to nearby *vs* distant nodes at each culture age, a 2-tailed Wilcoxon signed rank sum test was used. To compare the contribution of persistent links at each age, Friedman's rank test was used. Lastly, for the skewness of the link length distributions, a z-test was calculated based on the skewness estimate taken over the standard error of the skewness estimate. The P value was then calculated using the online statistics analysis tool (http://www.quantitativeskills.com/sisa/calculations/signhlp.htm, accessed November, 2010).

## Supporting Information

Figure S1 **Robustness of results to changes in link persistence threshold: Basic topological properties.** To check the influence of the persistent link definition threshold on the basic network statistics, results were calculated over a range of thresholds. The plots show results calculated from 10 trials (10 cultures), at a lower and higher link persistence threshold than the main results. Graphs on the left are from networks thresholded at 15% link-persistence (i.e. link presence required in at least 15% of network-wide bursts), and graphs on the right are from networks thresholded at 35% link-persistence. As for the main results, in cases where no links were found the data were excluded from the analysis, resulting in *n* of 6 to 10 for each age. At both 15% and 35% link-persistence thresholds there was a slight dip in the number of links between DIVs 14 and 21 (consistent with the 25% threshold results) and there was an increase in the number of links from DIV 21 onwards (again consistent with the main results). For all three link-persistence thresholds, the number of nodes fluctuated slightly between the ages. Edge density of the networks (second row) varied differently for each of the alternative link persistence thresholds. Moreover, at the 15% and 35% threshold levels some networks were overly dense –breaking the assumption of ‘sparseness’ required to assess ‘small-worldness’.
(PDF)

Figure S2 **Robustness of results to changes in link persistence threshold: Complex topological properties.** To check influence of the persistent link definition threshold on the complex network statistics, results were calculated over a range of thresholds. The graphs show mean path length, clustering coefficient and small-worldness (top, middle and bottom rows respectively) for the networks thresholded at 15%, 25% and 35% link-persistence. Results are from 10 trials (10 cultures), as for the main results, in cases where no links were found the data were excluded from the analysis, resulting in n of 6 to 10 for each age. Mean path length and clustering coefficient were normalized to the value expected for a random network. Small-worldness was calculated conservatively as (*C_real_*/*C_lattice_*)/(*L_real_*/*L_rand_*). Mean path length (top row) was relatively stable for all three thresholds, although at the 15% link-persistence threshold it increased slightly between DIV 28 and 35, this increase was not found to be significant (ANOVA P = 0.511). Clustering coefficient (second row) followed an increasing trend at all three thresholds. Small-worldness (bottom row) showed the same trend of increasing small-worldness between DIVs 14 and 28 at the 15% and 25% persistent link definition thresholds, however at the 35% threshold the edge density of the networks precluded accurate assessment of small-worldness.
(PDF)

Figure S3 **Robustness of small-world result: Validation of empirical results against those from random and lattice networks.** To check the robustness of the small-world result, complex network statistics from all three link-persistence thresholds were compared against the values expected for an equivalent lattice as well as those for an equivalent random network. Low, medium and high thresholds required link persistence in 15%, 25% and 35% of network-wide bursts respectively. Each graph shows the mean network statistic obtained from the real networks, against the value expected from an equivalent lattice network and the value expected from a population of equivalent random networks (same number of nodes and links in all cases). For all three thresholds the mean path length (first page of graphs) is close to that of a random network and less than that of a lattice. Likewise, for all three thresholds the clustering coefficient increased from close to the value expected from a random network, to close to the value expected for a lattice (second page of graphs).
(PDF)

Figure S4 **Global efficiency and conservative global-efficiency based ‘Small-Worldness’ of persistent networks.** To check the influence of disconnected networks on the measure of network integration, the global efficiency was tested (since mean path length is designed for connected networks and some of the networks were disconnected). Global efficiency is a measure of integration that is not affected by network dis-connectedness. The global efficiency was close to 1 at all ages, indicating a high level of integration and confirming the mean path length result. Moreover, the global efficiency-based small-worldness increased with culture age, consistent with the main results.
(PDF)

Figure S5 **Ability of the link definition threshold to identify genuine peaks.** A: To check whether the defined links (i.e. above link definition threshold) appeared to be genuine peaks in the cross covariance (XCov) plots, mean XCov plots were obtained on a per-channel basis. Plots are shown from a representative channel during a representative burst. The left hand plot shows the mean XCov value at each lag, from all channel pairs with a peak above the threshold (i.e. those that were considered to be related). There are two well-defined peaks and no obvious false positives. To check that genuine peaks were not missed, a mean XCov plot was obtained from all channel pairs with a peak≤threshold (right hand plot). There are no clear peaks. In addition to checking the mean plots, a number of individual XCov plots, over a range of cultures at each age, were manually inspected (i.e. checking for false positives or negatives). In all cases, those with a maximum peak above the threshold, appeared to contain a genuine peak in the plots, whilst those that did not cross the threshold showed no sign of peaks. B: To check the actual link definition thresholds used and see how they compared to the mean cross-covariance peak over all links, the mean XCov threshold for each age, was compared to the mean XCov peak for each age. Results are depicted in a bar chart, as can be seen, the mean XCov threshold is well above the mean XCov peak plus one standard deviation.
(PDF)

Figure S6 **Conservative small-worldness: guarding against high small-worldness values when clustering coefficient is low.** The first graph (Panel A) shows the mean path length, clustering coefficient and small-worldness values normalized against the expected values from a population of equivalent random networks. The second graph (Panel B) shows the raw network properties alongside those expected from equivalent random and lattice null hypothesis networks. At DIVs 14 and 21, small-worldness is met (Panel A) despite the clustering coefficient being far from the value expected for a lattice network (Panel B). It is not until after DIV 21 that the clustering coefficient approaches the value obtained for a lattice. Small-worldness is defined as L≥L_random_ and C>>C_random_ [73], and the small-world metric has been defined as: (C/C_rand_)/(L/L_rand_)>1 [74]. Therefore an overly optimistic small-world result can be obtained if the clustering coefficient of the random equivalent networks is very low, since normalized values of >>1 can be achieved despite a very low absolute clustering coefficient. Thus, whilst the cultures had a small-world metric >1 at DIVs 14 and 21 (left hand graph), it was not considered that these networks met the small-world criterion, (since their clustering coefficient was so low compared to a lattice). To address this issue, a conservative estimate of small-worldness based on: (C/C_Lattice_)/(L/L_random_) was used for the present study.
(PDF)

Table S1 **Mathematical definitions of the complex network measures used in this study.**
(PDF)

Text S1 **Selection and validation of statistical tests.**
(PDF)
