# Supplementary material for: Emergence of a Small-World Functional Network in Cultured Neurons
Source: PLoS Comput Biol. 2012 May 17;8(5):e1002522. doi: 10.1371/journal.pcbi.1002522 (PMC3355061; doi:10.1371/journal.pcbi.1002522)

Robustness of results to changes in link persistence threshold: Basic topological properties of the persistent networks as a function of culture age

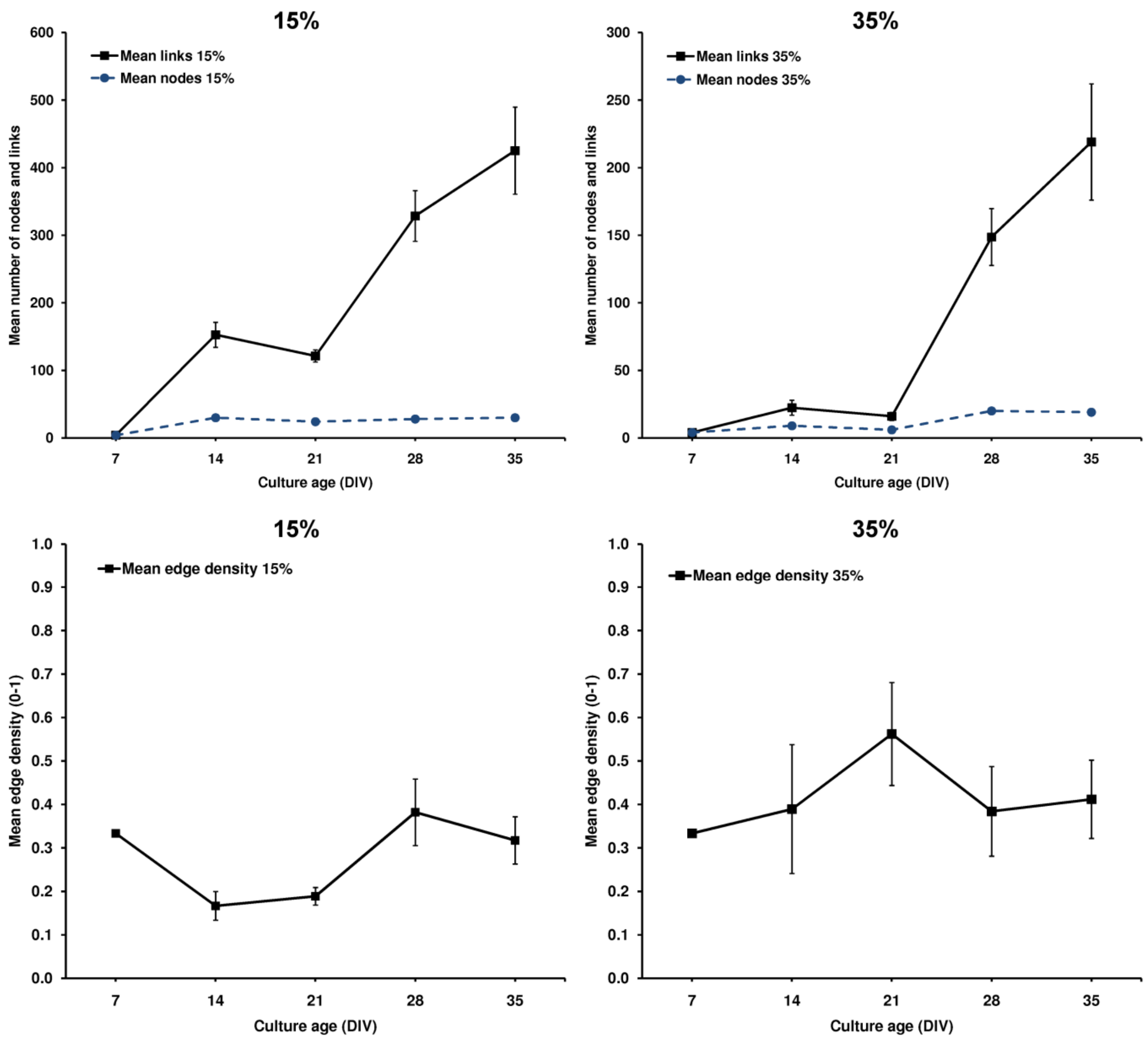

Supplement: Figure S1 — Robustness of results to changes in link persistence threshold: Basic topological properties. To check the influence of the persistent link definition threshold on the basic network statistics, results were calculated over a range of thresholds. The plots show results calculated from 10 trials (10 cultures), at a lower and higher link persistence threshold than the main results. Graphs on the left are from networks thresholded at 15% link-persistence (i.e. link presence required in at least 15% of network-wide bursts), and graphs on the right are from networks thresholded at 35% link-persistence. As for the main results, in cases where no links were found the data were excluded from the analysis, resulting in n of 6 to 10 for each age. At both 15% and 35% link-persistence thresholds there was a slight dip in the number of links between DIVs 14 and 21 (consistent with the 25% threshold results) and there was an increase in the number of links from DIV 21 onwards (again consistent with the main results). For all three link-persistence thresholds, the number of nodes fluctuated slightly between the ages. Edge density of the networks (second row) varied differently for each of the alternative link persistence thresholds. Moreover, at the 15% and 35% threshold levels some networks were overly dense –breaking the assumption of ‘sparseness’ required to assess ‘small-worldness’. (PDF) [file pcbi.1002522.s001.pdf]
