# Supplementary material for: Emergence of a Small-World Functional Network in Cultured Neurons
Source: PLoS Comput Biol. 2012 May 17;8(5):e1002522. doi: 10.1371/journal.pcbi.1002522 (PMC3355061; doi:10.1371/journal.pcbi.1002522)

Robustness of results to changes in link persistence threshold: Complex topological properties of the persistent networks as a function of culture age

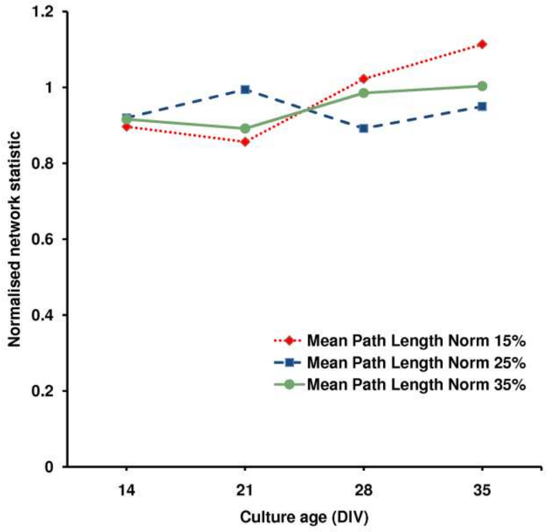

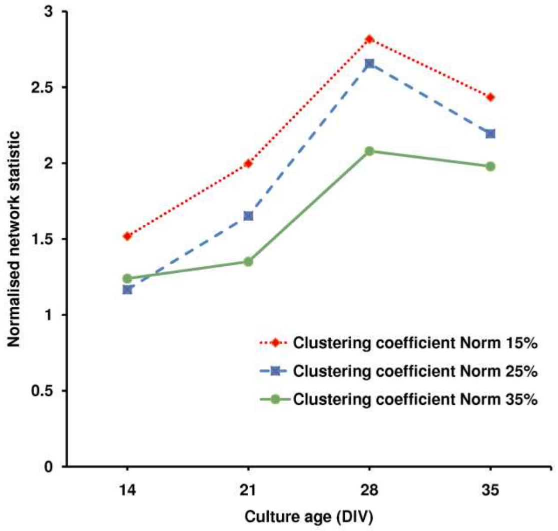

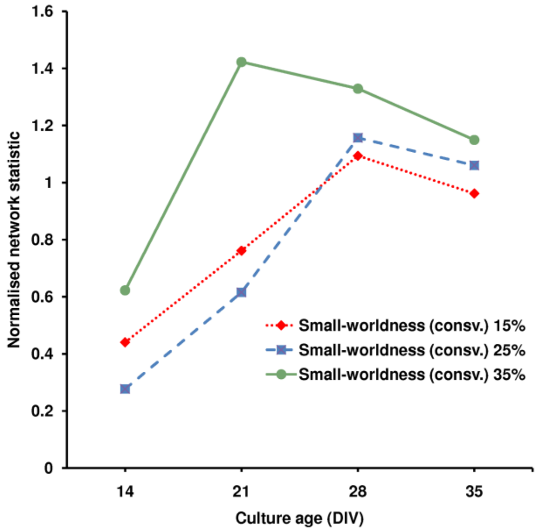

Supplement: Figure S2 — Robustness of results to changes in link persistence threshold: Complex topological properties. To check influence of the persistent link definition threshold on the complex network statistics, results were calculated over a range of thresholds. The graphs show mean path length, clustering coefficient and small-worldness (top, middle and bottom rows respectively) for the networks thresholded at 15%, 25% and 35% link-persistence. Results are from 10 trials (10 cultures), as for the main results, in cases where no links were found the data were excluded from the analysis, resulting in n of 6 to 10 for each age. Mean path length and clustering coefficient were normalized to the value expected for a random network. Small-worldness was calculated conservatively as (Creal/Clattice)/(Lreal/Lrand). Mean path length (top row) was relatively stable for all three thresholds, although at the 15% link-persistence threshold it increased slightly between DIV 28 and 35, this increase was not found to be significant (ANOVA P = 0.511). Clustering coefficient (second row) followed an increasing trend at all three thresholds. Small-worldness (bottom row) showed the same trend of increasing small-worldness between DIVs 14 and 28 at the 15% and 25% persistent link definition thresholds, however at the 35% threshold the edge density of the networks precluded accurate assessment of small-worldness. (PDF) [file pcbi.1002522.s002.pdf]
