# Supplementary material for: Emergence of a Small-World Functional Network in Cultured Neurons
Source: PLoS Comput Biol. 2012 May 17;8(5):e1002522. doi: 10.1371/journal.pcbi.1002522 (PMC3355061; doi:10.1371/journal.pcbi.1002522)
Supplement: Figure S3 — Robustness of small-world result: Validation of empirical results against those from random and lattice networks. To check the robustness of the small-world result, complex network statistics from all three link-persistence thresholds were compared against the values expected for an equivalent lattice as well as those for an equivalent random network. Low, medium and high thresholds required link persistence in 15%, 25% and 35% of network-wide bursts respectively. Each graph shows the mean network statistic obtained from the real networks, against the value expected from an equivalent lattice network and the value expected from a population of equivalent random networks (same number of nodes and links in all cases). For all three thresholds the mean path length (first page of graphs) is close to that of a random network and less than that of a lattice. Likewise, for all three thresholds the clustering coefficient increased from close to the value expected from a random network, to close to the value expected for a lattice (second page of graphs). (PDF) [file pcbi.1002522.s003.pdf]

Robustness of the small world result: Empirical network properties *vs* expected values from random and lattice null hypothesis networks

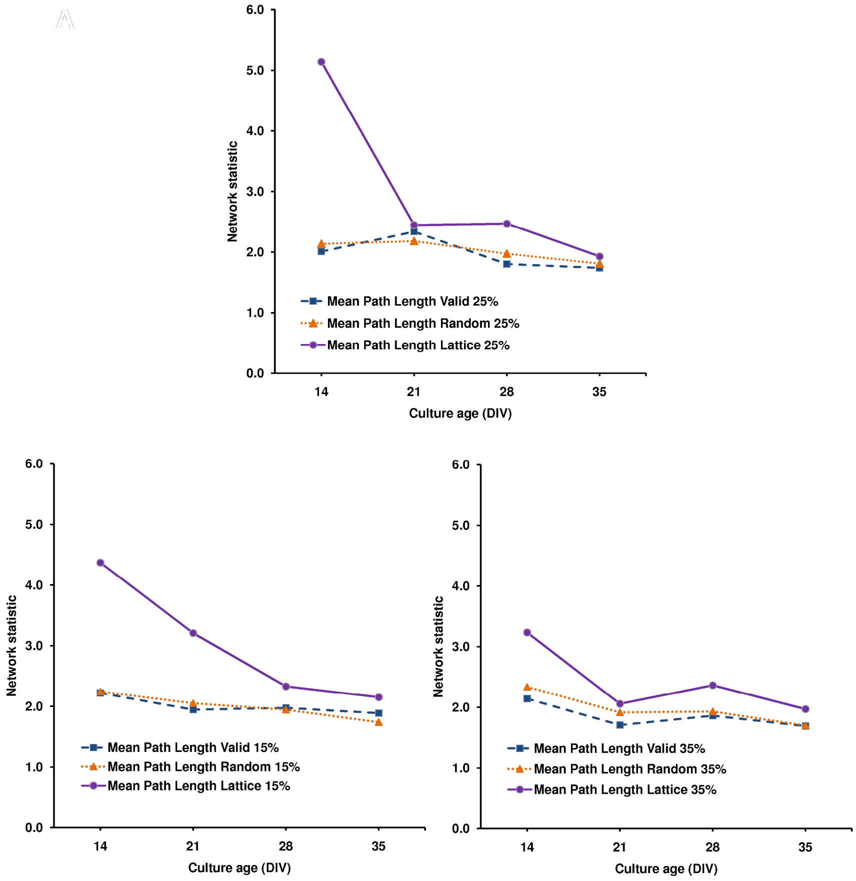

Mean path length is close to the value expected for a random network, regardless of persistent link definition threshold.

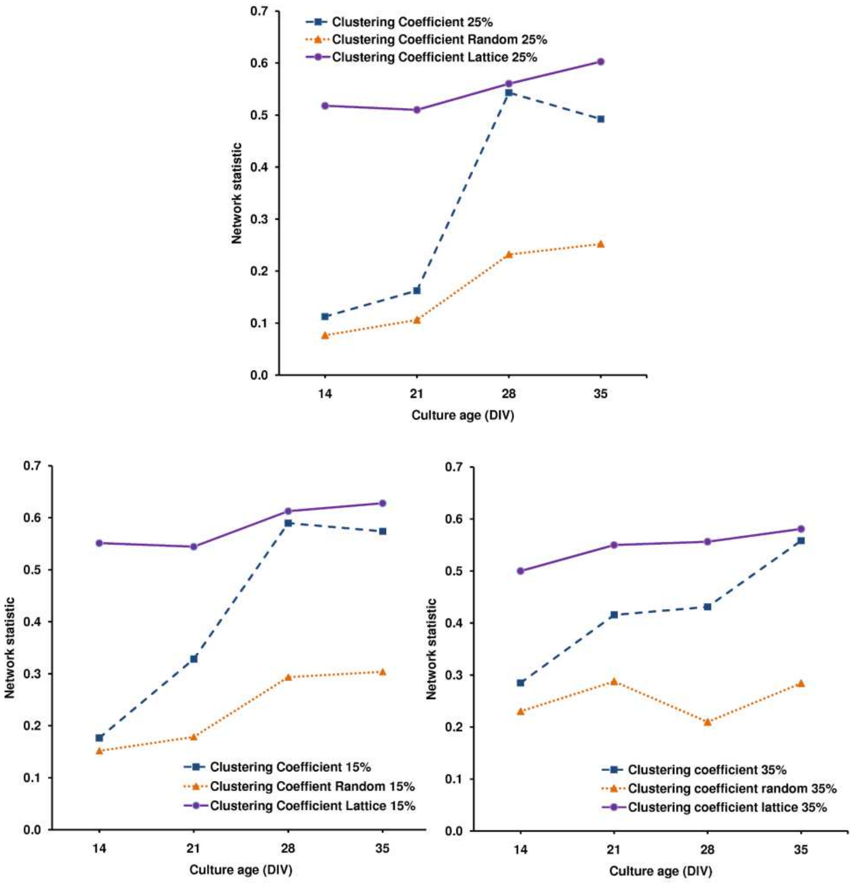

Clustering coefficient shows increasing trend towards the value expected for a lattice, regardless of persistent link definition threshold.
