# Supplementary material for: Emergence of a Small-World Functional Network in Cultured Neurons
Source: PLoS Comput Biol. 2012 May 17;8(5):e1002522. doi: 10.1371/journal.pcbi.1002522 (PMC3355061; doi:10.1371/journal.pcbi.1002522)
Supplement: Figure S4 — Global efficiency and conservative global-efficiency based ‘Small-Worldness’ of persistent networks. To check the influence of disconnected networks on the measure of network integration, the global efficiency was tested (since mean path length is designed for connected networks and some of the networks were disconnected). Global efficiency is a measure of integration that is not affected by network dis-connectedness. The global efficiency was close to 1 at all ages, indicating a high level of integration and confirming the mean path length result. Moreover, the global efficiency-based small-worldness increased with culture age, consistent with the main results. (PDF) [file pcbi.1002522.s004.pdf]

Global efficiency based small-worldness

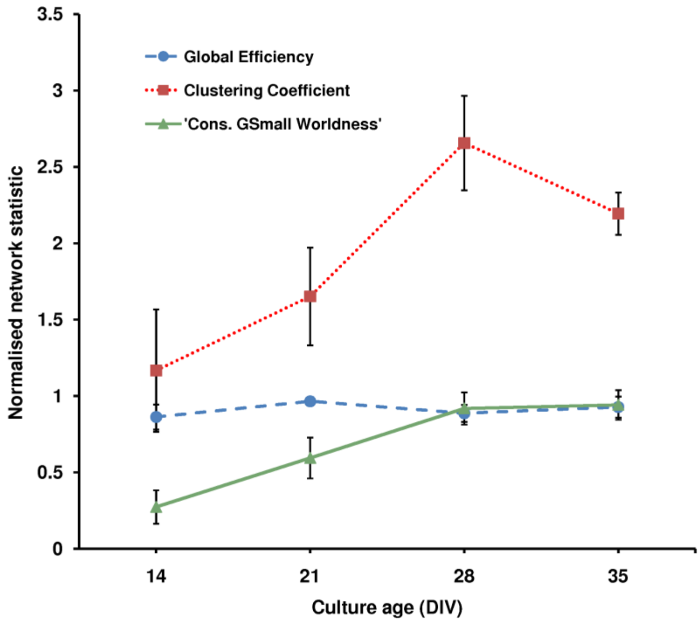

Global efficiency, clustering coefficient and conservative global-efficiency based 'small-worldness' of persistent networks as a function of culture age.

Averages (*n* = 4-6), were normalized as follows: Global efficiency (*E*) and clustering coefficient (*C*) were normalised against the expected value from an equivalent population of random networks (*n* = 50), therefore clustering coefficient is the same as in Figure 3 main text. Small-worldness was calculated conservatively as (C_{real} / $C_{lattice}$) / (E_{real}/E_{rand}). Error bars represent ± s.e.m. The global efficiency-based small-worldness increased with culture age (as per the mean path length based small-worldness).
