# Supplementary material for: Emergence of a Small-World Functional Network in Cultured Neurons
Source: PLoS Comput Biol. 2012 May 17;8(5):e1002522. doi: 10.1371/journal.pcbi.1002522 (PMC3355061; doi:10.1371/journal.pcbi.1002522)
Supplement: Figure S5 — Ability of the link definition threshold to identify genuine peaks. A: To check whether the defined links (i.e. above link definition threshold) appeared to be genuine peaks in the cross covariance (XCov) plots, mean XCov plots were obtained on a per-channel basis. Plots are shown from a representative channel during a representative burst. The left hand plot shows the mean XCov value at each lag, from all channel pairs with a peak above the threshold (i.e. those that were considered to be related). There are two well-defined peaks and no obvious false positives. To check that genuine peaks were not missed, a mean XCov plot was obtained from all channel pairs with a peak≤threshold (right hand plot). There are no clear peaks. In addition to checking the mean plots, a number of individual XCov plots, over a range of cultures at each age, were manually inspected (i.e. checking for false positives or negatives). In all cases, those with a maximum peak above the threshold, appeared to contain a genuine peak in the plots, whilst those that did not cross the threshold showed no sign of peaks. B: To check the actual link definition thresholds used and see how they compared to the mean cross-covariance peak over all links, the mean XCov threshold for each age, was compared to the mean XCov peak for each age. Results are depicted in a bar chart, as can be seen, the mean XCov threshold is well above the mean XCov peak plus one standard deviation. (PDF) [file pcbi.1002522.s005.pdf]

Ability of the link definition threshold to identify genuine peaks

A

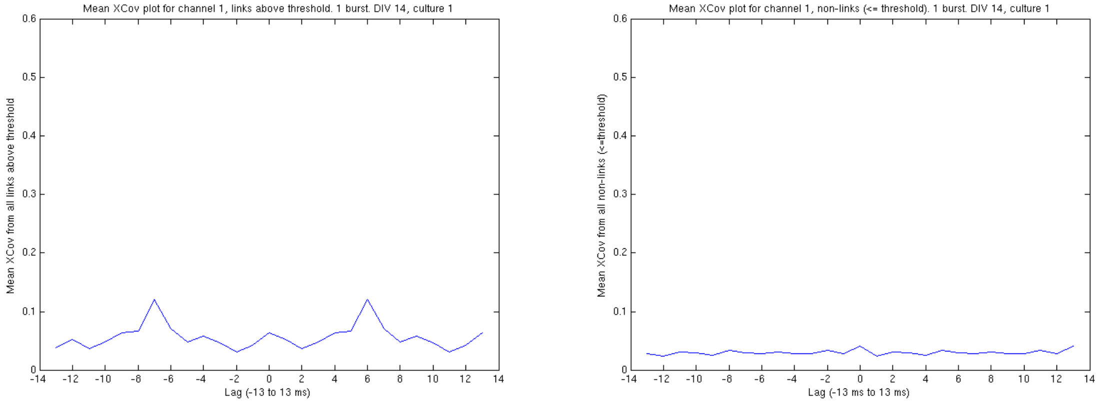

The link definition threshold detected genuine peaks in the cross covariance plot (left), sub-threshold cross covariance plots did not show evidence of missed links (right).

Mean XCov plots were obtained on a per-channel basis. The plots are from a representative channel during a representative burst. Each plot shows the mean XCov value at each lag. The left hand plot shows all channel pairs with a peak above the threshold (i.e. those that were considered to be related). The right hand plot shows all channel pairs with a peak ≤ threshold (i.e. those that were considered to be un-related). NOTE: the slight peak at zero lag in the right hand plot was excluded, since times < 1 ms were considered too short for synaptic transmission.

B

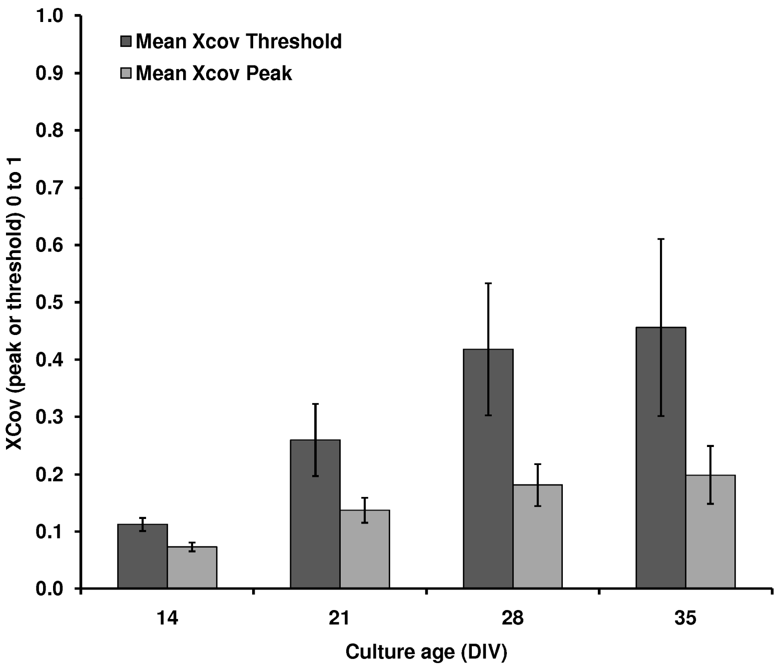

The mean XCov threshold and peak at each culture age: Mean XCov threshold is well above the mean XCov peak plus one standard deviation.

Mean cross covariance threshold (dark grey bars) and mean cross covariance peak (light grey bars) for all cultures at each stage of spontaneous development (4 conditions). Each bar was calculated from the mean XCov threshold (or peak), taken over all channel pairs, from all bursts (for each culture, then over all cultures). Error bars show the expected mean ± one standard deviation (i.e. the variability between XCov thresholds or peaks).
