# Supplementary material for: Emergence of a Small-World Functional Network in Cultured Neurons
Source: PLoS Comput Biol. 2012 May 17;8(5):e1002522. doi: 10.1371/journal.pcbi.1002522 (PMC3355061; doi:10.1371/journal.pcbi.1002522)
Supplement: Figure S6 — Conservative small-worldness: guarding against high small-worldness values when clustering coefficient is low. The first graph (Panel A) shows the mean path length, clustering coefficient and small-worldness values normalized against the expected values from a population of equivalent random networks. The second graph (Panel B) shows the raw network properties alongside those expected from equivalent random and lattice null hypothesis networks. At DIVs 14 and 21, small-worldness is met (Panel A) despite the clustering coefficient being far from the value expected for a lattice network (Panel B). It is not until after DIV 21 that the clustering coefficient approaches the value obtained for a lattice. Small-worldness is defined as L≥Lrandom and C>>Crandom [73], and the small-world metric has been defined as: (C/Crand)/(L/Lrand)>1 [74]. Therefore an overly optimistic small-world result can be obtained if the clustering coefficient of the random equivalent networks is very low, since normalized values of >>1 can be achieved despite a very low absolute clustering coefficient. Thus, whilst the cultures had a small-world metric >1 at DIVs 14 and 21 (left hand graph), it was not considered that these networks met the small-world criterion, (since their clustering coefficient was so low compared to a lattice). To address this issue, a conservative estimate of small-worldness based on: (C/CLattice)/(L/Lrandom) was used for the present study. (PDF) [file pcbi.1002522.s006.pdf]

Conservative small-worldness: Guarding against high small-worldness values when clustering coefficient is low

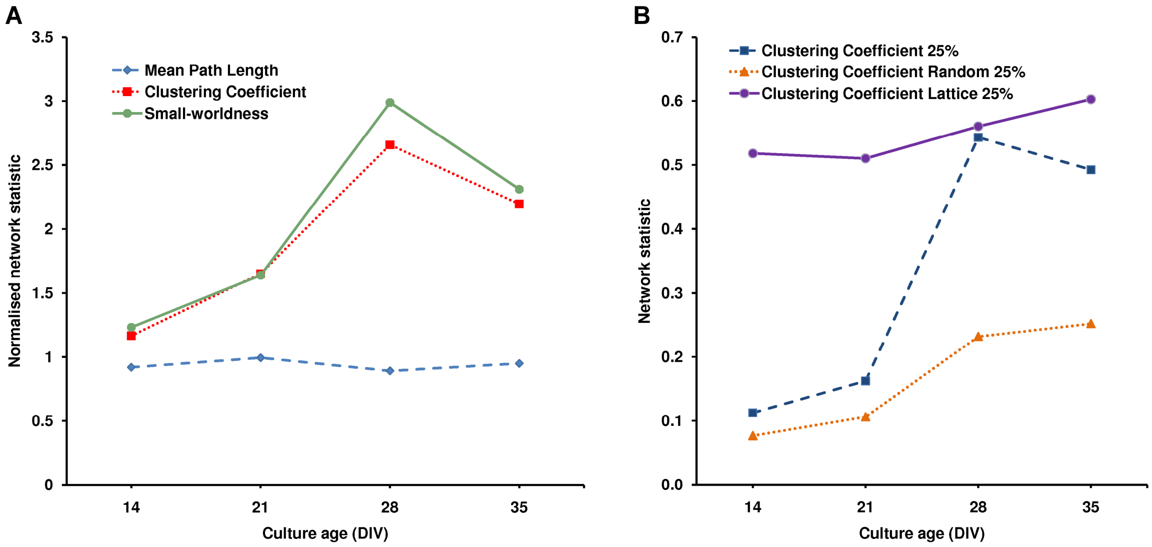

In some cases, small-worldness is met despite the clustering coefficient being far from the value expected for a lattice.

Panel A: All values are normalised against the expected value from a population of equivalent random networks (*n*=50) - as per the typical small-world calculation. Panel B: The raw (un-normalised) network properties are compared to those expected from equivalent random (*n*=50) and lattice networks.
