# Supplementary material for: Emergence of a Small-World Functional Network in Cultured Neurons
Source: PLoS Comput Biol. 2012 May 17;8(5):e1002522. doi: 10.1371/journal.pcbi.1002522 (PMC3355061; doi:10.1371/journal.pcbi.1002522)
Supplement: Table S1 — Mathematical definitions of the complex network measures used in this study. (PDF) [file pcbi.1002522.s007.pdf]

Table S1: Mathematical definitions of complex network statistics used in the present study

Ref.	Nomenclature	Description of use
N/A	n	Number of nodes (All networks were calculated on the basis of 59 possible nodes, corresponding to the 59 recordable electrodes). The set of nodes in the network is denoted N (this is all nodes for which links were found), and the number of nodes n.
N/A	m	Number of links (edges) in the network (all networks were calculated on the basis of 59 * 58 (3422) possible links (a node was not allowed to have a link to itself). Networks compared were directed symmetrical; all links were counted in both directions to avoid ambiguity with directed links. The set of links in the network is denoted M, and the number of links found m† (each counted twice).
N/A	ξ	Edge density of the network (the same as 'cost' used in [8] is the number of edges found compared to the maximum possible number of edges (given the number of nodes in the networks). For the symmetrical networks used herein, this was calculated as: $$m / n(n\text{-}1)$$
[14]	a_{ij}	The connection status of nodes *i,j*, $a_{ij} = 1$ when a link exists between nodes *i,j,*(they are said to be neighbours). As the networks compared were symmetrical, $a_{ij} == a_{ji}$.
	k_i	Degree of node i is the number of neighbors of the node, calculated as: $$k_i = \sum_{j \in N} a_{ij}$$ As nodes must have at least 1 link to be considered part of the network, k_i is always > 0

Ref.	Nomenclature	Description of use
[14]	d_{ij}	Shortest path length (distance) between nodes i and j, calculated as:<br>$d_{ij} = \sum_{a_{uv} \in g_{i \leftrightarrow j}} a_{uv}$<br>Where $g_{i \leftrightarrow j}$ is the shortest path (geodesic) between *i* and *j*. For all disconnected pairs *i,j*, $d_{ij} = \infty$
	l_i	The path length of node *i* is the sum of the shortest distance to all other nodes (normalized to *n*-1), where shortest distance is calculated as:<br>$l_i = 1/n\ \Sigma\ (\ \Sigma\ j\ d_{ij} / n\text{-}1)$<br>Where *j* is for each other node in the network.
[16]	c^{ws}_{i}	Following Watts and Strogatz (ws), the clustering coefficient of node *i* is the number of edges between the neighbours of *i* divided by the maximum possible number of edges. This was calculated as:<br>$C_i = m / k_i (k_i\text{-}1)$<br>Where *m* is the number of edges between the neighbours of *i*.
[14]	*L*	Mean path length, taken over all node pairs between which a path exists (i.e. $d_{ij} \neq \infty$), calculated as:<br>$L = 1/n\ \Sigma\ l_i$<br>Where l_i is the average distance between node *i* and all other nodes.
	C	Mean clustering coefficient, taken over all nodes in the network, calculated as:<br>$C = 1/n\ \Sigma\ c_i$
	K	Mean node degree, taken over all nodes in the network, calculated as:<br>$K = 1/n\ \Sigma\ k_i$

Ref.	Nomenclature	Description of use
[14, 64]	E	Following Latora *et al.* [64], global efficiency is the average inverse shortest path length, taken over all nodes in the network, calculated as: $E = 1/n\, \Sigma\, E_i\ = 1/n\, \Sigma\, (\, \Sigma\, j\, d_{ij}^{-1} / n\text{-}1)$
[64]	$C^{ws}{}_{Lattice}$	Expected Watts and Strogatz Clustering Coefficient for an equivalent lattice network, calculated as: $C^{ws} = ¾(k – 2) /(k – 1)$
[14]	$C^{ws}{}_{Rand}$	Expected Watts and Strogatz Clustering Coefficient for an equivalent random network (calculated from a population of random networks with the same number of nodes and links: see section on generation of equivalent null hypothesis networks).
	L_{Rand}	Expected mean shortest path length for an equivalent random network (calculated from a population of random networks with the same number of nodes and links: see section on generation of equivalent null hypothesis networks).
[17]	S^{ws} =	Small Worldness. Calculated as: $(C^{ws}/C^{ws}{}_{Rand}) / (L/ L_{Rand})$ Using global efficiency instead mean shortest path length [8]: $(C^{ws}/C^{ws}{}_{Rand}) / 1/(E/ E_{Rand})$
N/A	CS^{ws} =	Conservative Small Worldness. Calculated as: $(C^{ws}/C^{ws}{}_{Lattice}) / (L/ L_{Rand})$ Using global efficiency instead mean shortest path length [8]: $(C^{ws}/C^{ws}{}_{Lattice}) / 1/(E/ E_{Rand})$

† NOTE, there is sometimes ambiguity in the notation used for definitions of complex network statistics, with *l* referring to path length and to number of links, here *l* is used to refer to path length and *m* for the number of links.
