# Supplementary material for: Emergence of a Small-World Functional Network in Cultured Neurons
Source: PLoS Comput Biol. 2012 May 17;8(5):e1002522. doi: 10.1371/journal.pcbi.1002522 (PMC3355061; doi:10.1371/journal.pcbi.1002522)
Supplement: Text S1 — Selection and validation of statistical tests. (PDF) [file pcbi.1002522.s008.pdf]

Text S1: Selection and validation of statistical tests for the present study

The statistical tests applied to the properties analysed in the present study were selected based on the experiment design and form of the resultant data.

Assumptions of tests used:

Data were evaluated to ensure they met the assumptions of the proposed statistical test. The assumptions for all tests used are detailed below.

One-way ANOVA (Analysis of Variance):

- Homogeneity of variances
- Data from a normal distribution
- More than two groups

Kruskal-Wallis:

- The population variances are comparable
- Data from a non-normally distributed population
- More than two groups

Two-tailed Wilcoxon signed ranks test:

- Paired samples (i.e. two observations per subject)

Friedman's Rank Test:

- Multiple observations per subject

Z-test:

- Data from a normal distribution
- Two groups

Levene Test for equality of variances:

- Data from an approximately normal distribution*
- There must be at least 3 observations from one or more of the populations

*The Levene test is less sensitive to departures from normality than most 'homogeneity of variance' tests .

Notes on evaluation of data against assumptions:

All data used for the present study met the assumption of independence within groups – i.e. cultures were only included once in each experimental group.

Random selection of data: The experiment design (see Introduction) and analysis decisions (see cell cultures and sample population selection in Methods) facilitated random selection of sample data; use of data from multiple generations ensured that results were not biased by the characteristics of an individual generation.

Independence of samples between groups: For the main complex network statistics (mean path length, clustering coefficient and small-worldness), the samples for each developmental stage were treated as independent, since only a subset of the cultures used were suitable for complex network analysis (see calculation of network statistics in Methods). This meant that multiple observations per subject could not be applied to these properties. For the remaining network properties, data were also treated as independent samples, since subsets of the cultures were typically different between ages. The exception was the link persistence values, where the data for the contribution of persistent links at each age were treated as multiple observations per subject.

Normality was tested using Shapiro-Wilk normality test. Additionally, box plots were visually inspected, and measures of the mean, median, skewness and kurtosis were used to evaluate deviation from normality. In cases where non-normality was suspected statistics were re-calculated using non-parametric test equivalents. In all cases, the non-parametric test results followed the same trend as those reported in the manuscript.

Homogeneity of variances was tested using the Levene statistic

Assessment of data distribution and selection of statistical tests:

Normality was verified using the Shapiro Wilk normality test, however, since n numbers were small, data were also verified for normality by inspection of box plots and comparison of the mean *vs* median values.

Together, these were used to determine the distribution of the data and the appropriate statistical test. A subset of the data verification is provided below.

<u>Example 1a: Normally distributed data</u>

Mean number of nodes

DIV 14: n=6, Mean: 26.33, SD: 10.01, StdErr: 4.088, Median: 22.50

KS: P>0.05, SW: 0.7880

DIV 21: n=5, Mean: 18.00, SD: 5.788, StdErr: 2.588, Median: 18.00

KS: P>0.05, SW: 0.7620

DIV 28: n=6, Mean: 22.50, SD: 8.526, StdErr: 3.481, Median: 22.50

KS: P>0.05, SW: 0.7880

DIV 35: n=5, Mean: 28.00, SD: 8.631, StdErr: 3.860, Median: 29.00

KS: P>0.05, SW: 0.7620

Where: SD = standard deviation of the mean, StdErr = standard error of the mean,

KS = Kolmogorov Smirnoff normality test result.

SW = Shapiro Wilk normality test result.

Box plots were visually inspected to check the data range and variability (see Box plots).

Comparison of the mean *vs* median values did not suggest deviation from normality, neither did results from the normality tests. Therefore, the data were deemed to be ~normal. Since the Levene statistic demonstrated that data did not break the assumption of homogeneous variances (P=0.565) and there were multiple experimental groups (4 DIVs), a one-way ANOVA was selected.

Box plots for mean number of nodes

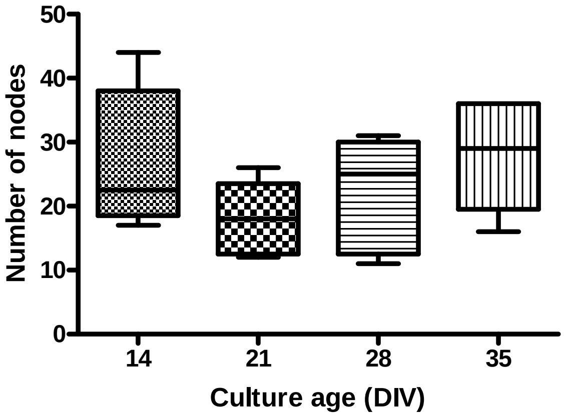

Example 1b: Normally distributed data

Conservative small-worldness

DIV 14: n=6, Mean: 0.2773, SD: 0.2569, StdErr: 0.1049, Median: 0.2728

KS: P>0.05, SW: 0.7879

DIV 21: n=5, Mean: 0.6154, SD: 0.2843, StdErr: 0.1272, Median: 0.4886

KS: P>0.05, SW: 0.7620

DIV 28: n=6, Mean: 1.157, SD: 0.1030, StdErr: 0.04204, Median: 1.165

KS: P>0.05, SW: 0.7879

DIV 35: n=5, Mean: 1.060, SD: 0.1028, StdErr: 0.04595, Median: 1.062

KS: P>0.05, SW: 0.7620

Where: SD = standard deviation of the mean, StdErr = standard error of the mean,

KS = Kolmogorov Smirnoff normality test result.

SW = Shapiro Wilk normality test result.

Box plots were visually inspected to check the data range and variability (see Box plots).

Comparison of the mean *vs* median values did not suggest deviation from normality, neither did results from the normality tests. Therefore, the data were deemed to be ~normal. Since there were multiple experimental groups (4 DIVs), a one-way ANOVA was selected. However, since the Levene statistic revealed that homogeneity of variances was violated, the Brown Forsythe F ratio and Welsh F ratio were also assessed for significance. In both cases, results followed the same trend as those calculated under the assumption of homogeneous variances.

Box Plots for conservative small-worldness

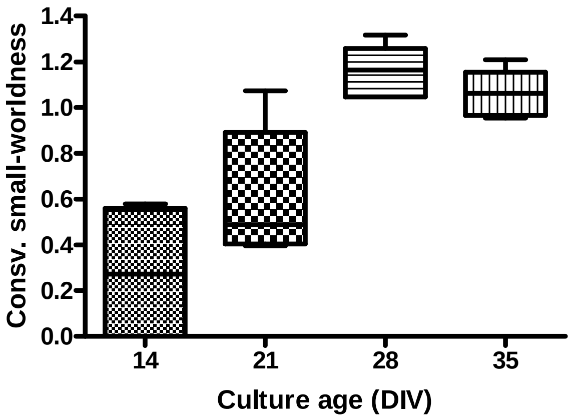

Example 2: Non-normally distributed data

Median burst propagation time (5TH to 95TH Percentile data)

DIV 14: n=6, Mean: 0.6443, SD: 0.6654, StdErr: 0.2717, Median: 0.3891

KS: P<0.05 (0.0160)

DIV 21: n=8, Mean: 0.3872, SD: 0.2904, StdErr: 0.1027, Median: 0.2753

SW: 0.0402, KS: P>0.05

DIV 28: n=7, Mean: 0.1034, SD: 0.01879, StdErr: 0.007101, Median: 0.1083

SW: 0.4542, KS: P>0.05

DIV 35: n=6, Mean: 0.1078, SD: 0.03836, StdErr: 0.01566, Median: 0.1116

SW: KS: P>0.05

Where: SD = standard deviation of the mean, StdErr = standard error of the mean,

SW: = Shapiro Wilk normality test result, KS = Kolmogorov Smirnoff normality test result.

Box plots were visually inspected to check the data range and variability (see Box plots).

DIV 14 was significantly different from normal (P=0.0160), therefore a non-parametric test was required and since the data were independent samples, the Mann-Whitney U-test was selected.

Box plots for median burst propagation time

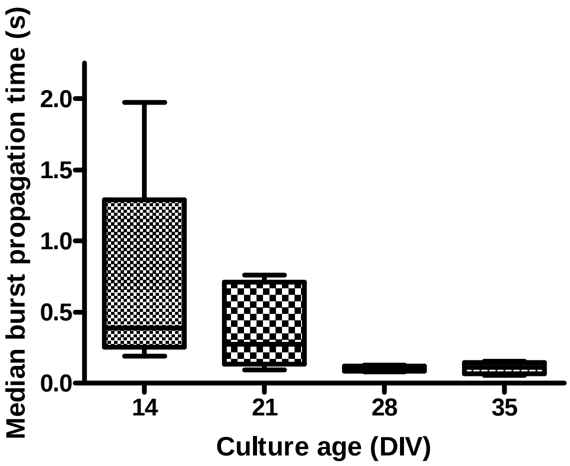

Log Scale:

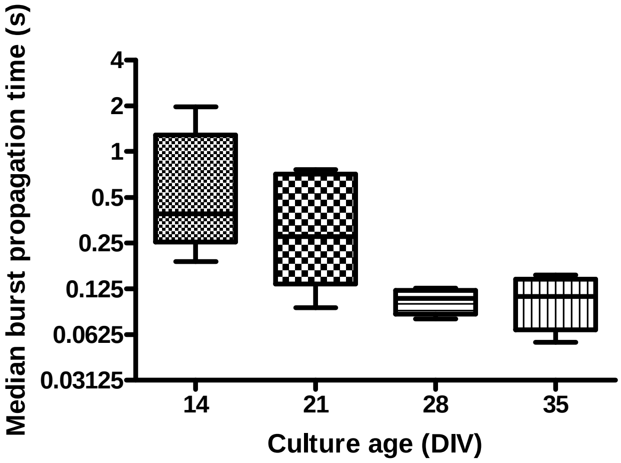

Verification of statistical power:

Minimum sample size was established for detecting nominal differences in the means with 80% statistical power (at P=0.05 significance level) (Whitley and Ball 2002). The nominal difference was set at 2 times standard deviation of the mean. This level merely established the minimum number of samples required to detect relatively large differences between experimental groups.

To achieve ≥80% statistical power for detecting a difference of 2 times standard deviation of the mean, at the P = 0.05 significance level requires the following n numbers.

- t-test (difference in the means), n = 4,
- Mann-Whitney U-test, n = 4 (based on negligible reduction in statistical power compared to the t-test in cases where sample size is <10 (Lord 1950)).
- One-way ANOVA, n = 7.
- Kruskal-Wallis, n = 7.

In cases, where sample size is lower, then statistical power will be reduced and there is an increased risk of false negatives (i.e. type 2 errors). For example, with n = 6, the statistical power of a one-way ANOVA to detect the nominal difference in the means is reduced to 75% power, and with n = 5, it reduces to 64% power. The consequence of this is that it is more difficult to detect significant results and smaller differences between the properties of the experimental groups may be missed. However, for the present study, the non-significant findings were typically the result of genuinely small differences in the properties (i.e. differences < 1 standard deviation, for example number of nodes, short mean path length). Therefore, the available statistical power was deemed to be sufficient.

Equations for the t-test were from (Whitley and Ball 2002). For the ANOVA, an online calculator was used (http://euclid.psych.yorku.ca/cgi/power.pl)

References

Lord, E. (1950). "Power of the modified t-Test (u-Test)." Biometrika 37(1/2): 64-77.
Whitley, E. and J. Ball (2002). "Statistics review 4: sample size calculations." Crit Care. 6(4): 335-41 Epub 2002 May 10.
